# Ecological roles of dominant and rare prokaryotes in acid mine drainage revealed by metagenomics and metatranscriptomics

**DOI:** 10.1038/ismej.2014.212

**Published:** 2014-11-07

**Authors:** Zheng-Shuang Hua, Yu-Jiao Han, Lin-Xing Chen, Jun Liu, Min Hu, Sheng-Jin Li, Jia-Liang Kuang, Patrick SG Chain, Li-Nan Huang, Wen-Sheng Shu

**Affiliations:** 1 State Key Laboratory of Biocontrol, Key Laboratory of Biodiversity Dynamics and Conservation of Guangdong Higher Education Institutes, College of Ecology and Evolution, Sun Yat-sen University, Guangzhou, PR China; 2 Metagenomics Applications Team, Genome Science Group, Los Alamos National Laboratory, Los Alamos, NM, USA

## Abstract

High-throughput sequencing is expanding our knowledge of microbial diversity in the environment. Still, understanding the metabolic potentials and ecological roles of rare and uncultured microbes in natural communities remains a major challenge. To this end, we applied a ‘divide and conquer' strategy that partitioned a massive metagenomic data set (>100 Gbp) into subsets based on K-mer frequency in sequence assembly to a low-diversity acid mine drainage (AMD) microbial community and, by integrating with an additional metatranscriptomic assembly, successfully obtained 11 draft genomes most of which represent yet uncultured and/or rare taxa (relative abundance <1%). We report the first genome of a naturally occurring *Ferrovum* population (relative abundance >90%) and its metabolic potentials and gene expression profile, providing initial molecular insights into the ecological role of these lesser known, but potentially important, microorganisms in the AMD environment. Gene transcriptional analysis of the active taxa revealed major metabolic capabilities executed *in situ*, including carbon- and nitrogen-related metabolisms associated with syntrophic interactions, iron and sulfur oxidation, which are key in energy conservation and AMD generation, and the mechanisms of adaptation and response to the environmental stresses (heavy metals, low pH and oxidative stress). Remarkably, nitrogen fixation and sulfur oxidation were performed by the rare taxa, indicating their critical roles in the overall functioning and assembly of the AMD community. Our study demonstrates the potential of the ‘divide and conquer' strategy in high-throughput sequencing data assembly for genome reconstruction and functional partitioning analysis of both dominant and rare species in natural microbial assemblages.

## Introduction

Microorganisms are critical to the functioning of virtually all ecosystems on our planet (Harris, 2009; Jiao *et al.*, 2010), yet we know little about their precise ecological and functional roles in the community (Prosser *et al.*, 2007). This hurdle is mainly caused by the high biodiversity of most microbial assemblages (Tiedje, 1994) and the uncultivable properties of the majority of microbes from the environment (Pace, 1997). With the benefit of cultivation-independent metagenomics approaches, recent studies have successfully reconstructed the genomes of dominant members in the communities (Tyson *et al.*, 2004; Jones *et al.*, 2011; Mason *et al.*, 2012), and advanced our understanding of the metabolic potentials and functional significance of microbes *in situ*. However, natural microbial communities are typically composed of a few dominant species followed by a large number of rare taxa (Sogin *et al.*, 2006). These low-abundance organisms may be representatives of novel microbial lineages (Castelle *et al.*, 2013; Kantor *et al.*, 2013) and play crucial roles in biogeochemical cycles and overall metabolic fluxes (Musat *et al.*, 2008; Wrighton *et al.*, 2012). Moreover, the evenness patterns of low-abundance taxa are important in defining microbial ecosystem dynamics (Huber *et al.*, 2007). Nevertheless, the nature and complexity of information in metagenomic data sets and insufficient sequencing depth and computing resources make it difficult to capture the genomic information and ecological roles of low-abundance populations (Sharon *et al.*, 2013). Furthermore, metagenomics provides no information concerning the dynamic expression and regulation of genes in the environment. Recently, metatranscriptomics approaches have been used to reveal the community-wide gene expression profiles and ecophysiology of natural microbial assemblages (Hewson *et al.*, 2009; Ottesen *et al.*, 2011). However, direct analyses of both DNA and RNA sequence pools from the same communities are few (for example, Frias-Lopez *et al.*, 2008; Shi *et al.*, 2009; Stewart *et al.*, 2011), although coupled community genomic and transcriptomic analysis has the potential to discover and characterize the relative transcriptional levels of a large number of genes (Frias-Lopez *et al.*, 2008), and unravel the functional diversity and ecological partitioning in microbial communities.

**Figure 1:**
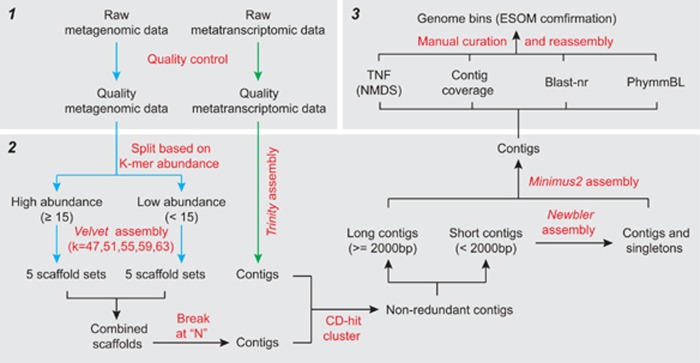
A schematic flow chart depicting the ‘divide and conquer' strategy for the genome assembly with combined metagenomic and metatranscriptomic data. Quality control of raw data (1), sequence assembly (2) and genome binning (3) are shown. ESOM, emergent self-organizing map; NMDS, Non-metric Multidimensional scaling; TNF, tetranucleotide frequency. See details in the section of Materials and methods.

Acid mine drainage (AMD) environments have great advantages for the study of microbial community structure and function because of its biological and geochemical simplicity (Baker and Banfield 2003; Denef *et al.*, 2010). Mine tailings represents a major source of AMD via microbially mediated oxidative dissolution of sulfide minerals. The tailings of Fankou Pb/Zn mine in Guangdong, South China, has been intensively studied to reveal the phylogenetic and functional dynamics of microbial communities in the tailings acidification and AMD generation processes (Huang *et al.*, 2011; Chen *et al.*, 2013). As a routine analysis of an ongoing survey, the microbial composition of the AMD sample collected in September of 2012 (Table 1) was assessed using 16S rRNA gene cloning and sequencing (see Supplementary methods). The results showed the Bacteria and Archaea domains of the AMD community was, respectively, dominated by unclassified *Betaproteobacteria* (*Bacteria*) and *Euryarchaeota* (*Archaea*), with several other rare taxa (Supplementary Figure 1). To reveal the ecological roles and functional partitioning (that is, different functions performed by different taxa) of AMD taxa in the community, a novel pipeline was developed to reconstruct genomes for both the dominant and rare taxa from the metagenomic and metatranscriptomic data containing enormous number of read pairs (outlined in Figure 1), and the *in situ* transcriptional profiles of the active populations were analyzed.

## Materials and methods

### Sampling, physicochemical and community diversity analysis

Sample collection and physicochemical analysis were conducted as previously described (Kuang *et al.*, 2013 and as detailed in Supplementary Methods). Experimental procedures for DNA and RNA extraction, rRNA subtraction, RNA amplification and complementary DNA (cDNA) synthesis are described in Supplementary Methods. Microbial diversity was analyzed by cloning and sequencing PCR-amplified 16S rRNA genes (Supplementary methods). Sequences were compared with those in the Ribosomal Database Project. Barcoded 454 pyrosequencing targeting the hypervariable V4 region of 16S rRNA genes was also conducted to evaluate the microbial community structure. Raw pyrosequencing data were processed and analyzed as previously described (Kuang *et al.*, 2013; for details see Supplementary Methods).

### Metagenomic and metatranscriptomic sample preparation, sequencing and *de novo* assembly

For metagenomic sequencing, two libraries with insert sizes of 500 bp and 2000 bp were independently generated from the total community genomic DNA sample. For metatranscriptomic sequencing, a library with an insert size of 300 bp was generated from the cDNA sample. The three libraries were pair-end sequenced (2 × 101 bp) on an Illumina Hiseq 2000 instrument (Illumina, Macrogen Inc., Seoul, Korea), producing approximately 105 and 8.7 Gbp of sequences from the DNA and cDNA sample, respectively. The raw reads were quality filtered as detailed in Supplementary Methods and the results are shown in Supplementary Table 1. To reduce the extremely high memory consumption during assembly of high-throughput sequencing-based metagenomic data (Hess *et al.*, 2011), a ‘divide and conquer' strategy was used to partition the quality metagenomic reads into low- and high-abundance K-mers groups according to the K-mers depth using Khmer (version 0.3; Pell *et al.*, 2012; depth: 15, K-mer: 31; reads with length less than 63 bp were removed). The high- and low-abundance K-mer reads were then, respectively, assembled at a range of K-mers (47, 51, 55, 59 and 63; Supplementary Table 2) using Velvet (version 1.1.06; Zerbino and Birney, 2008). In addition, *de novo* transcriptome reconstruction was conducted for the quality metatranscriptomic data using Trinity (Grabherr *et al.*, 2011). The 10 scaffold sets generated by the paired-ended assembly using Velvet were combined and broken into contigs by one or more continuous ‘N', to stringently avoid potential chimeric scaffolds. Although this step might break valid connections in the scaffolds, we did so to remove duplicated contigs from multiple assembly in the next step, and also to benefit for the gap closing of continuous Ns. These contigs were then pooled with the contig set from the metatranscriptomic assembly with Trinity. CD-hit algorithms (Li and Godzik, 2006) were applied to cluster the contigs into sequence families (options: −c 0.98, −aS 1, −g 1 and –r 1), for the removal of duplicated contigs from multiple assembly. The resulting non-redundant contigs were then sorted into two pools based on their length, then Newbler and Minimus2 were used for assembly as previously reported (Mason *et al.*, 2012). Contigs with length <2000 bp were assembled using Newbler to generate longer contigs (options: −consed, −mi 98 and −ml 40). The singletons and contigs obtained from the Newbler assembly were combined with the non-redundant contigs with length ⩾2000 bp, and further assembled based on their overlap using Minimus2 (http://sourceforge.net/apps/mediawiki/amos/index.php?title=Minimus2) with a minimum percentage cutoff of 98%.

### Genome binning and genome annotation

Both supervised and unsupervised approaches were used for (and to improve the accuracy of) genome binning (Strous *et al.*, 2012). For supervised classification, contigs from the Mininus2 assembly with length ⩾3000 bp (28.9 Mb in total) were compared with the National Center for Biotechnology Information (NCBI) non-redundant (nr) protein database using BLASTx (*e*-value ⩽10^−5^). These contigs were also compared against nearly 1900 complete genomes in the NCBI RefSeq database (Pruitt *et al.*, 2007) and nine AMD draft genomes (Tyson *et al.*, 2004; Baker *et al.*, 2006, 2010) via phymmBL program (Brady and Salzberg, 2009). For unsupervised classification, tetranucleotide frequency (TNF) was first calculated for all contigs ⩾3000 bp. Then TNF-based Hierarchical Agglomerative Clustering using Euclidean Distance and *ward* criterion was performed for the contigs. To determine the best number of bins for grouping the contigs, Non-metric Multidimensional scaling analysis based on the contigs' TNF distance matrix was conducted. This resulted in a total of 11 bins (Supplementary Figure 2). Within each bin, contigs with a divergent phylogenetic assignment or coverage were manually removed or merged into another bin. Subsequently, potential chimeric contigs in each bin were detected by BLASTn against themselves. As most of the putative chimeras were from repeat regions, the contigs in each bin were shredded into 500 bp overlapped fake reads and reassembled using phrap (de la Bastide and McCombie, 2007). Finally, 11 draft genomes were obtained and the nucleotide sequences were deposited at MG-RAST under the accession numbers of 4565622.3−4565632.3. Emergent self-organizing map-based analysis was conducted for genome binning validation as previously described (Dick *et al.*, 2009). The completeness of the draft genomes was estimated as previously described (Hess *et al.*, 2011). For genome annotation, protein-coding genes were predicted for each draft genome using Genemark (Zhu *et al.*, 2010). Functional annotation of the predicted genes was conducted based on BLASTx analysis (*e*-value⩽10^−5^) against the proteins in the databases of NCBI-nr, Kyoto Encyclopedia of Genes and Genomes (KEGG) and evolutionary genealogy of genes: Non-supervised Orthologous Groups (eggNOG). The matched genes were then assigned to KEGG orthologs (KOs), KEGG pathways, KEGG categories, clusters of orthologous groups of proteins (COG) catalogs and COG categories for further analysis. Details of all analyses are given in the Supplementary Methods.

### Statistical analyses

Relative transcriptional activity (RTA) of a given gene, or KO, or COG from a genome was calculated in a normalized way as:


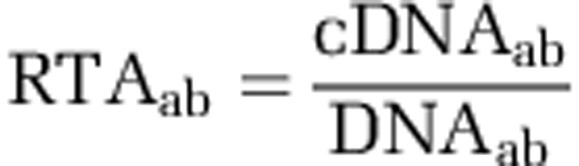


where cDNA_ab_ is the relative abundance of cDNA reads matching gene (or KO or COG) a in genome b, and DNA_ab_ is the relative abundance of DNA reads matching gene (or KO or COG) a in genome b. The relative abundance was calculated as the percentage of cDNA (or DNA) reads matching gene (or KO or COG) a in genome b dividing by the total cDNA (or DNA) reads in genome b.

For each of the selected genomes, KOs with cDNA reads counts >1.5 times the interquartile range of cDNA read counts of all the KOs in the genome were labeled as an expression outliers (Gifford *et al.*, 2013). Then the expression outliers from all genomes were combined to make a table of outlier orthologous relationships. The indicator value (IV) of each expression outlier was calculated as:


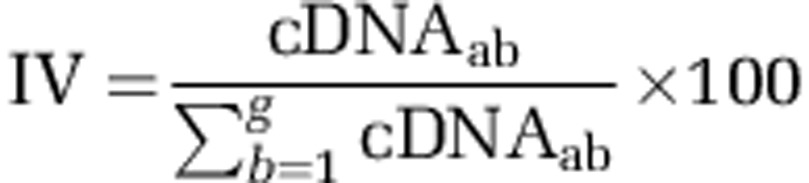


where cDNA_ab_ was calculated as the number of cDNA reads matching the expression outlier KO a in genome b dividing by the total number of cDNA reads in genome b, *g*=number of genomes. Those KO labeled as expression outliers and with an IV>50 were identified as indicator KO (Gifford *et al.*, 2013).

Statistically significant differences in relative transcriptional activity of genes (or COG or KEGG category) across AMD taxa were determined using the non-parametric Wilcoxon rank-sum test (*P*<0.05).

## Results

### Physicochemical characteristics and microbial community composition and diversity of the AMD sample

**Figure 2:**
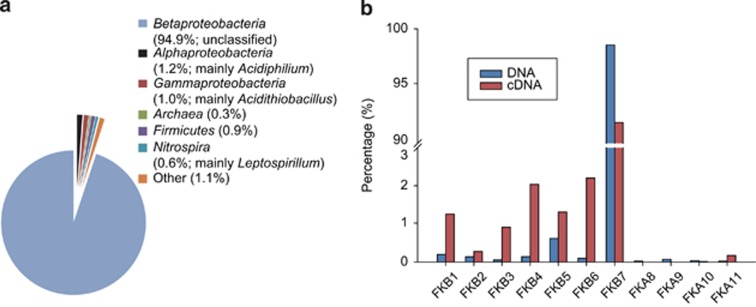
Relative abundance of AMD taxa as revealed by (**a**) 16S rRNA genes analysis based on 454 pyrosequencing, and (**b**) unassembled quality metagenomic and metatranscriptomic reads. See Table 2 for the most closely related organisms for the 11 AMD taxa, and see Supplementary Table 3 for the details of quality reads for each taxon. The relative abundance of a given populations was calculated as the number of quality DNA reads mapped to its corresponding draft genome divided by the total number of quality DNA reads.

The AMD sample was characterized with low pH (2.6) and total organic carbon and high levels of ferric iron, sulfate and heavy metals, such as Zn, Pb, Cu, Co, Cd and Cr (Table 1). Pyrosequencing analysis of 16S rRNA genes revealed a microbial community of very low diversity, with a total of 81 operational taxonomic units from 10 845 quality sequences assigned to *Bacteria* (99.7%) and *Archaea* (0.3% Figure 2a). *Proteobacteria* (*Betaproteobacteria,* 94.9% *Alphaproteobacteria*, 1.2% *Gammaproteobacteria*, 1.0%)*, Firmicutes* and *Nitrospira,* respectively, accounted for 97.1%, 0.9% and 0.6% of the community. These results were in accordance with that revealed by 16S rRNA gene clone library analysis (Supplementary Figure 1). Moreover, mapping the quality metagenomic reads to the operational taxonomic units representative sequences also identified a similar population structure of the AMD community (Figure 2a and Supplementary Figures 1 and 3).

### Genome reconstruction and phylogeny

With the benefit of the ‘divide and conquer' assembly strategy using metagenomic data, and by integrating with a separate metatranscriptomic assembly (Figure 1), a total of 11 draft genomes were successfully reconstructed, of which 10 were rare members (relative abundance<1%) of the AMD community (Table 2, Figure 2b and Supplementary Figure 4). The taxonomic information of the genomes was then evaluated. Owing to the absence of 16S rRNA gene sequences for seven of the draft genomes (Table 2), a phylogenetic tree was built based on a concatenation of 31 universal and rarely horizontally transferred protein-coding marker genes occurring in 658 fully sequenced genomes in the STRING database (Szklarczyk *et al.*, 2011; 1133 genomes initially, with only one genome in each genus selected for the phylogenetic tree construction) and the 11 draft genomes (Supplementary Figure 5). Taxonomic affiliation of the draft genomes was also evaluated by BLASTx analysis against the NCBI-nr database and phymmBL analysis of their contigs. The results showed that FKB1 and FKB2 belonged to *Acidithiobacillus* and were most similar to *At. ferrooxidans* and *At. thiooxidans*, respectively. FKB3 and FKB4 were closely related to *Leptospirillum rubarum* (group II) and *L. ferrodiazotrophum* (group III), respectively. FKB5 was related to *Acidiphilium cryptum*, and FKB6 was most similar to *Alicyclobacillus acidocaldarius*. The unclassified FKB7 harbored a complete 16S rRNA gene sequence sharing 96% similarity with that of *Ferrovum myxofaciens* strain P3G (Johnson *et al.*, 2014; Supplementary Figure 6). The archaeal genomes of FKA8, FKA9, FKA10 and FKA11 were most closely related to that of *Picrophilus torridus*, *Candidatus Micrarchaeum acidiphilum* ARMAN-2, *C. Parvarchaeum acidiphilum* ARMAN-4 and *C. Parvarchaeum acidophilus* ARMAN-5, respectively. Based on genomic alignment analysis using MUMmer (Delcher *et al.*, 2003), a good matching of nucleotide sequences between the draft genomes and their references was identified (Supplementary Figure 7), suggesting the validity of the assembly and genome binning processes. The taxonomic composition of the AMD community based on the 11 draft genomes was comparable to those revealed by 16S rRNA gene-based analyses (Figure 2a and Supplementary Figures 1 and 3), except for the absence of ARMAN 16S rRNA genes which could not be amplified by the universal primers used (for example, 27F/1492R, 515F/806R). Among the 11 draft genomes, FKB1 and FKB2, FKB3 and FKB4, FKA9 and FKA10 were from the genus of *Acidithiobacillus*, *Leptospirillum* and *C. Parvarchaeum acidiphilum*, respectively. To distinguish their genomic information from the contigs pool, we used the combined information including coverage, TNF and BLAST searches to known databases as detailed in Materials and methods and Supplementary Information. For the three pairs of taxa, genome coverages were 58 vs 37, 19 vs 40 and 42 vs 26, and average GC contents were 58.0% vs 53.2%, 57.7% vs 59.0% and 35.9% vs 39.9%, respectively. Bidirectional BLAST analysis revealed that 1690, 1631 and 979 ortholog genes were shared between FKB1 and FKB2, FKB3 and FKB4, FKA9 and FKA10, respectively, and the identities relative to each other were 63%, 71% and 66% on average based on amino-acid sequence similarity. Comparable similarity values at the amino-acid level have been reported between *At. ferrooxidans* and *At. thiooxidans* (about 69%, Valdés *et al.*, 2008, 2011) and the acidophilic archaea ARMAN-4 and ARMAN-5 (about 71%, Baker *et al.*, 2010). In contrast, FKB3 and FKB4 showed a higher similarity than that previously reported between known *Leptospirillum* spp. (Goltsman *et al.*, 2009), with an average amino-acid identity over 55%.

### Global analysis of metabolic potentials and gene expression

To determine the metabolic potentials and gene expression of taxa in the AMD community, gene prediction and functional annotation were conducted for the 11 draft genomes. A range of 1208–3011 genes were predicted from the genomes, of which over 73%, 65% and 64% matched the genes in the NCBI-nr, KEGG and eggNOG databases, respectively (Supplementary Table 3). The gene sets of the 11 taxa were dominated by those involved in the KEGG categories of amino acid, carbohydrate, energy and nucleotide metabolism, and translation (Supplementary Figure 8). At the finer KEGG metabolic pathway level, different patterns of expressed genes were shown toward different taxa. Many central pathways, including oxidative phosphorylation, amino-acid biosynthesis, pyrimidine and purine metabolism, nitrogen metabolism and carbon metabolism, dominated the transcript pools (Supplementary Figure 9). Other pathways like two-component regulatory systems and ATP-binding cassette transporters were also highly expressed, indicating that the AMD taxa conducted active response and adaptation to the changing environment.

### Gene expression profiles of active taxa

To further reveal the ecological roles of active taxa in the AMD community, the 10 genes with the highest relative transcriptional activity in each taxon were characterized (Table 3). As previously reported in marine microbial communities (Gifford *et al.*, 2013), there was a significant positive relationship between the expression level of a KO in a specific taxon and how commonly it was harbored in the other taxa (Supplementary Figure 10; Wilcoxon rank-sum test, *P*<0.05), suggesting that the highly expressed genes were more likely to be shared across multiple taxa. For this reason, the indicator genes representing the processes garnering the most transcriptional effort by that taxon were also characterized. The top 10 indicator genes with the highest relative abundance in the transcript pool of each taxon are shown in Supplementary Table 4. This analysis excluded FKA8, FKA9 and FKA10 due to the low occurrence of associated information in the metatranscriptomic data set (Supplementary Table 3).

**Figure 3:**
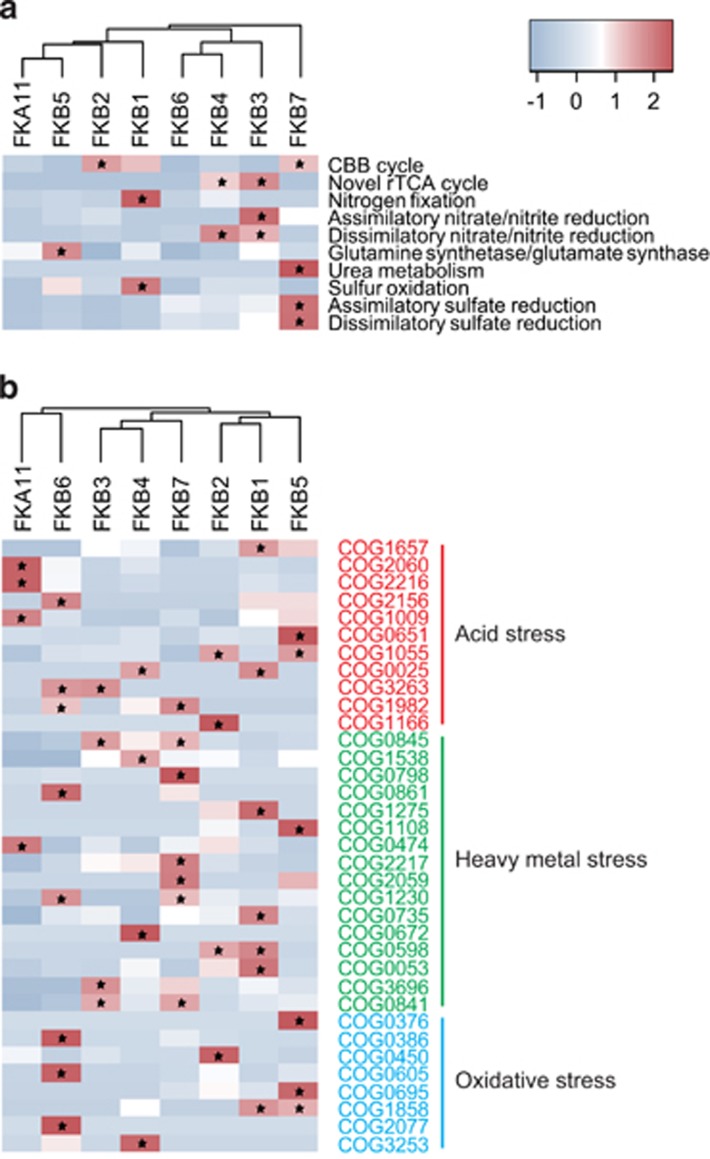
Profiling of (**a**) energy-related metabolisms and (**b**) COGs relevant to acid, heavy metal and oxidative stress in the eight transcriptionally active AMD taxa. The metabolisms and COGs are clustered based on their relative transcriptional activity (*z*-score normalized across all taxa, indicated by the color key). The relative transcriptional activity of a given metabolism is represented by the average number of relative transcriptional activity of all KOs assigned to this metabolism. Asterisk stands for significant overrepresentation of metabolism or COG in the taxon, determined by non-parametric Wilcoxon rank-sum test (*P*<0.05).

The AMD taxa should harbor multiple stress resistance mechanisms to deal with the extreme environmental conditions (for example, low pH and high levels of heavy metals). Transcriptional analysis revealed the expression of enzymes involved in the survival strategies for the resistance of low pH (Figure 3b and Supplementary Table 5), including (i) harboring a highly impermeable cell membranes to protons (hopanoid biosynthesis; COG1657), (ii) generating an inside-positive membrane potential through the uptake of potassium (COG2060, COG2216, COG2156), (iii) pumping out protons using Na^+^/H^+^ or K^+^/H^+^ antiporters, (iv) harboring cytoplasmic buffer molecules capable of sequestering protons (for example, lysine, histidine, arginine, H_3_PO_4_) and (v) degrading organic acids (Baker-Austin and Dopson, 2007). Similarly, the genes associated with heavy metals resistance, including those for Zn, Fe, Pb, Cd, Co, Cu and other heavy metals (Table 1), were also actively expressed in the AMD taxa. Oxidative stress represents another obstacle for survival in AMD environments (Ram *et al.*, 2005). Although no associated parameters were directly determined in this study, the expression of peroxiredoxin, superoxide dismutase and catalase genes indicated such a stress for the analyzed AMD community. On the other hand, chaperones like Hfq, DnaK, GroEL and HSP20 associated with repair of DNA and protein damage caused by the extreme conditions, were fairly highly represented in most of the bacterial taxa (Table 3).

#### *FKB1 and FKB2*

The *At. ferrooxidans*-like FKB1 and *At. thiooxidans*-like FKB2 were likely autotrophic for genes encoding the key enzymes involved in Calvin-Benson-Bassham carbon fixation cycle, including RuBisCO and *prkB* were expressed (Supplementary Table 6). The nitrogen fixation gene of *nifH* was highly expressed in FKB1 (Supplementary Tables 4 and 6). Both taxa harbored transcripts associated with glutamine synthetase for ammonium assimilation, and nitrate reductases for dissimilatory functions were highly expressed in FKB2 (Supplementary Tables 4 and 6). Both FKB1 and FKB2 could potentially oxidize sulfur as indicated by the expression of genes encoding sulfite-quinone reductase, tetrathionate hydrolase and thiosulfate:quinone oxidoreductase, whereas sulfur oxidation multienzyme complex (Sox, including *SoxAX*, *SoxB* and *SoxYZ*) system and sulfur dioxygenase were only expressed in FKB2 (Supplementary Tables 4 and 6). The highly expressed rusticyanin in FKB1 may indicate its activity in iron oxidation (Bonnefoy and Holmes, 2012; Supplementary Table 6). Notably, the high occurrence of transcripts associated with motility in FKB2, including genes for flagellar assembly and chemotaxis, suggested a much higher active motility than any other taxa (Supplementary Table 4).

#### FKB3 and FKB4

The *Leptospirillum-*affiliated FKB3 and FKB4 may perform carbon fixation via the novel reductive tricarboxylic acid cycle (Levic**á**n *et al.*, 2008; Goltsman *et al.*, 2009; Table 3 and Figure 3a). The expression of *nifHDK* genes in the *Leptospirillum* group III-like FKB4 may indicate an important role in nitrogen fixation as FKB1 (Goltsman *et al.*, 2009; Supplementary Table 4). The expression of *nirD* in FKB3 indicated a potential activity of dissimilatory nitrite reduction. Although *Leptospirillum* species were reported to oxidize sulfur (Johnson and Hallberg, 2008), the sulfate assimilation function was active in both FKB3 and FKB4 for thiamine biosynthesis, and the RuBisCO-like genes identified in these two taxa may also indicate an involvement in sulfur metabolism (Ashida *et al.*, 2005). The ferrous iron oxidation-associated genes encoding cytochrome 572 (cyt_572_) and cytochrome 579 (cyt_579_) were expressed in FKB3 and cyt_572_ expressed in FKB4 (Supplementary Table 6; Ram *et al.*, 2005). Indicator gene analysis showed that two families of two-component regulatory systems were overexpressed in the FKB4 (Table 3).

#### FKB5

The FKB5 was most closely related to *A. cryptum*, which has been found to grow on the small amounts of organic carbon originating from chemoautotrophic acidophilic iron/sulfur-oxidizers (Johnson and Hallberg, 2008), and reduce ferric iron coupled to the oxidation of glucose (Küsel *et al.*, 2002). Transcriptional analysis indicated a heterotrophic lifestyle of FKB5, with the expression of various transporting protein-coding genes for dissolved organic carbon resources (Supplementary Table 7). The organic carbon degradation-associated genes like formate dehydrogenase and branched-chain amino-acid aminotransferase, were among the most highly expressed (Table 3), and those for propionyl-CoA synthase and acetyl-CoA synthetases were also expressed. Ammonium assimilation may serve as an important strategy for nitrogen resource of FKB5 as evidenced by the detection of *nrgA*, *gltB* and *gltD* in the transcript pool (Supplementary Table 6). Most of the heterotrophic *Acidiphilium* spp. can oxidize reduced sulfur compounds for energy generation, while showing no nutritional requirement for them (Johnson and Hallberg, 2008). This was likely the case in the *A. cryptum*-like FKB5 as indicated by the expression of sulfur dioxygenase.

#### FKB6

The FKB6 showed the highest similarity to *A. acidocaldarius*, a thermoacidophilic heterotroph capable of using multiple sugars as carbon energy source (Lauro *et al.*, 2006). Genes related to sugar and polysaccharide transporters were expressed in FKB6 (Supplementary Table 7), and it devoted more transcriptional effort to phosphate acquisition (via phosphate ATP-binding cassette transporter) than any other taxa (Supplementary Table 4). Genes in the two-component system chemotaxis family, which respond to chemical stimuli by regulating the chemotactic sensitivity and extending the range of signal transduction, were also highly expressed (Supplementary Table 4).

#### FKB7

**Figure 4:**
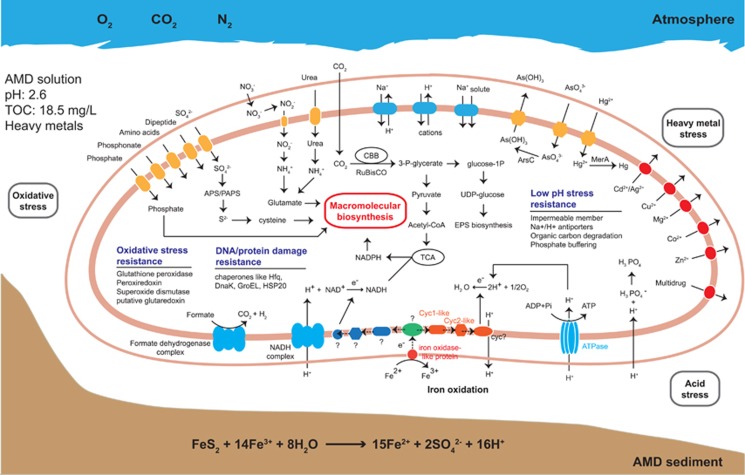
Metabolic abilities of *Ferrovum*-like FKB7 based on the expressed genes, which were predicted from its draft genome assembled from the metagenomic and metatranscriptomic data. Those associated with carbon fixation, nitrogen metabolism, assimilatory sulfur reduction, amino-acid biosynthesis, energy metabolism, transporters, stress response and putative iron oxidation pathway are shown.

The most dominant member of the community FKB7 (relative abundance>90%, Figure 2b) was determined to be *Ferrovum* spp. (Supplementary Figure 11), a newly discovered group within *Betaproteobacteria* that has been suggested to be widely distributed iron oxidizers (Hallberg *et al.*, 2006; Kuang *et al.*, 2013; Johnson *et al.*, 2014). The RuBisCO and *prkB* genes for carbon fixation were highly expressed in FKB7 (Figure 4 and Supplementary Table 6). No nitrogen fixation-associated genes were detected, whereas the expression of a urease (encoded by ureDABJCEFG operon), and nitrate transporter and assimilatory nitrate reduction genes indicated that this predominant taxon may use urea and nitrate as alternative nitrogen resources (Supplementary Table 6). The expression of *nirB* and *nirD* indicated a potential activity for dissimilatory nitrite reduction. Notably, sulfate reduction genes were highly expressed in an energy-consuming assimilatory pathway (Figure 4), which could provide reduced sulfur for the synthesis of cysteine and methionine and a range of other metabolites. Moreover, the expression of oxidative phosphorylation-associated genes was the highest among the eight taxa (Supplementary Figure 9). The two-component signal transduction system was enriched in the FKB7 transcript pool (KdpD-KdpE; Supplementary Table 4), which might be involved in pH stress through activating the K^+^ transporters for K^+^ influx to inhibit H^+^ influx. Besides, Na^+^/H^+^ antiporters and arginine decarboxylase may also be used for acid stress, and several genes encoding heavy metal exporting proteins and those for oxidative stress were also expressed (Figure 3b and Supplementary Table 5), indicating multiple stress mechanisms used for FKB7 to adapt to the extreme AMD conditions (Table 1).

To investigate the iron oxidation pathway of FKB7, we searched the draft genome for homolog genes involved in iron oxidation as reported in other iron oxidizers (Bonnefoy and Holmes, 2012) as previously conducted (Liljeqvist *et al.*, 2013), including *cyc*1/*rus*/*cyc*2 in *At. ferrooxidans*, *iro* in *At. ferrivorans*, *cox* operon in *Thiobacillus prosperus*, *fox* gene cluster in *Sulfolobus metallicus*, *foxEYZ* operon of *Rhodobacter capsulatus* SB1003, Cyt_572_/Cyt_579_ in *Leptospirillum* spp., *PioAB* in *Rhodopseudomonas palustris* TIE-1, and *MtrAB* in *Shewanellas* spp. and *Geobacter* spp. Two genes of FKB7 were similar to *cyc*1 (encoding cytochrome c552; 34% in amino-acid similarity and 84% in query coverage) and *cyc*2 (encoding a high-molecular mass cytochrome; 27% in amino-acid similarity and 97% in query coverage; Supplementary Figure 12), both are involved in the iron oxidation in *Acidithiobacillus* spp. Moreover, in the same contig with the *cyc*1-like gene, we identified a gene with relatively low-sequence similarity to the iron oxidase *iro* in *At. ferrooxidans* and *At. ferrivorans* (32% and 26% in amino-acid similarity, 85% and 92% in query coverage), respectively. All the three putative iron oxidation genes were highly expressed in FKB7 (Figure 4 and Supplementary Table 6). As such, this predominant taxon may gain electrons from ferrous iron via the iron oxidase-like protein and transfer them to the inner membrane through cytochromes encoded by *cyc*1- and *cyc*2-like genes (Figure 4).

#### FKA11

FKA11, the only active archaeon detected in the AMD system, was closely related to *Picrophilus torridus*, which is well known for its ability to live at pH around 0 and harbors a smaller genome size than any other nonparasitic aerobic microorganisms growing on organic substrates (Futterer *et al.*, 2004). Our indicator gene analysis revealed the genes involved in transcription and replication dominated the transcript pool, including DNA polymerase, RNA polymerase and factors for elongation, transcription and replication (Supplementary Table 4), indicating that FKA11 may be at a rapid growth stage. The central carbon metabolism may also be active as suggested by the gene expression of isocitrate dehydrogenase, a key enzyme in TCA cycle. This corresponded to the expression of several transporters for organic carbon resources, such as oligopeptide, dipeptide, amino acid and sugar (Supplementary Table 7), which are important for heterotrophic *P. torridus*-like species (Futterer *et al.*, 2004). The acid stress resistance was likely conducted by uptake of K^+^, using Na^+^/H^+^ antiporters and also organic acid degradation, whereas peroxiredoxin and thioredoxin reductase (nicotinamide adenine dinucleotide phosphate) genes for the response of oxidative stress were also expressed (Table 3 and Supplementary Table 5), guaranteeing the physiological activity of FKA11 in such an extreme environment (Table 1; Ciaramella *et al.*, 2005).

## Discussion

### ‘Divide and conquer' strategy for high-throughput sequencing data analysis and genome reconstruction of dominant and rare taxa

Both dominant and less dominant species are important to the overall function and dynamics of microbial communities (Huber *et al.*, 2007; Musat *et al.*, 2008; Denef *et al.*, 2010). Understanding the potential ecological roles of the rare (and often uncultured) taxa has been particularly challenging, however. Although this issue could now be explored through omics technologies with increased sequencing depth, such approaches are hampered by the subsequent assembling of large short-read sequence data sets (Sharon *et al.*, 2013). To overcome this, several recent studies have attempted targeted genome sequencing of rare taxa after specific enrichment using cell prefiltering and/or biostimulation technologies or single-cell isolation (Wrighton *et al.*, 2012; Castelle *et al.*, 2013; Kantor *et al.*, 2013; Rinke *et al.*, 2013). Although these elegant works have expanded our understanding of the phylogenetic characteristics and functional significance of these lesser known microbes in the environment, only a small fraction of the community is captured in the analyses. To gain a relatively comprehensive look at the community gene content and expression, and to reveal the functional roles of both the dominant and rare taxa in a single community, a naturally low-diversity microbial assemblage from an extreme AMD environment was selected in this study and subjected to parallel metagenomics and metatranscriptomics sequencing, generating a sequence data set of over 110 Gbp. Although lower computing memory assembler like IDBA_ud (Peng *et al.*, 2012) has been used to assemble large metagenomic data sets (Castelle *et al.*, 2013; Hug *et al.*, 2013; Kantor *et al.*, 2013), it was not feasible in our case because of the relatively limited computing resources (512 Gb RAM). Therefore, we attempted a novel ‘divide and conquer' strategy using velvet in the assembly of our short-read metagenomic data set, and the results were integrated with a separate assembling of the community cDNA sequences. This has enabled a successful reconstruction of 11 draft genomes, which represent both dominant and rare and/or uncultured taxa, allowing subsequent exploration of their ecological roles and functional partitioning in the AMD community.

### Genome construction and gene expression of naturally occurring Ferrovum *spp*.

Molecular investigations have documented that bacteria affiliated with the recently discovered genus ‘*Ferrovum*' are ubiquitous and thus likely play an important role in various AMD environments (Hallberg *et al.*, 2006; González-Toril *et al.*, 2011; Kuang *et al.*, 2013; Johnson *et al.*, 2014). Despite their wide distribution and high vitality in acidic mine waters, the isolation and cultivation of *Ferrovum* spp. have been difficult, indicating the extremely fastidious nature of these lesser known *Betaproteobacteria* (Johnson *et al.*, 2014). To date, only one pure laboratory isolate (*Ferrovum myxofaciens* strain P3G; Johnson *et al.*, 2014) and several mixed cultures (Hedrich *et al.*, 2009; Kimura *et al.*, 2011) have been reported, and genomic and gene expression information of naturally occurring *Ferrovum* spp. or laboratory isolates are lacking, precluding a comprehensive understanding of their metabolic potentials and *in situ* gene dynamics, which may provide insights into their ecological success in the environment. By deeply sequencing an AMD community predominated by unclassified *Betaproteobacteria*, we reconstructed the first genome of a natural *Ferrovum* population (that is, multiple individuals of FKB7; Figure 4, Table 2 and Supplementary Figure 11). Phylogenetic analysis based on 16S rRNA genes showed that FKB7 was similar to the few cultivated *Ferrovum* representatives (Supplementary Figure 6), and distantly related to the genus of *Nitrosospira* as previously indicated (Johnson *et al.*, 2014). Our transcriptional analysis combining the metagenomic and metatranscriptomic data indicated that the *Ferrovum*-like FKB7 could fix carbon via Calvin-Benson-Bassham cycle in the oligotrophic AMD with a low total organic carbon level, and conduct ferrous iron oxidation for energy generation with multiple putative electron transporting proteins (Figure 4). Thus, our study provides the first field evidence at the transcriptomic level for the assumption that these moderately acidophilic bacteria are obligately autotrophic and capable of growth only by ferrous iron oxidation (Rowe and Johnson, 2008; Hallberg, 2010; Johnson *et al.*, 2014). Surprisingly, although the *F. myxofaciens* strain P3G appears to be diazotrophic (Johnson *et al.*, 2014), the nitrogen fixation genes *nifHDK* were absent from the FKB7 genome (Figure 4), and urea and nitrate may instead serve as alternative nitrogen resources as evidenced by the expression of associated genes. These results indicated the high diversity and different evolutionary history of *Ferrovum* spp. Most previous attempts to isolate *Ferrovum* spp. into pure cultures failed because of the contamination of *Acidiphilium* spp. (Hedrich *et al.*, 2009; Kimura *et al.*, 2011; Johnson *et al.*, 2014), which could degrade the organic substances that are otherwise toxic to *Ferrovum* spp. Thus, it is possible that the co-occurring *Acidiphilium*-affiliated FKB5 may facilitate the dominance of FKB7 in the analyzed AMD community (Figure 2).

### Ecological roles and functional partitioning of AMD taxa

The phylogenetically distinct taxa in the AMD community showed different ecological roles with specific transcriptional behaviors to coexist in the harsh environment (Table 3 and Supplementary Tables 4 and 5). The availability of carbon and nitrogen resources is vital to the microbial communities populating the oligotrophic and nitrogen-limited AMD systems (Johnson and Hallberg, 2008). The *Acidithiobacillus*-like FKB1 and FKB2 and *Ferrovum*-like FKB7 could fix carbon using the Calvin-Benson-Bassham cycle (Johnson and Hallberg, 2008; Johnson *et al.*, 2014), whereas the *Leptospirillum*-like FKB3 and FKB4 may perform carbon fixation via the novel reductive tricarboxylic acid cycle (Goltsman *et al.*, 2009). In contrast, no carbon fixation genes were expressed in FKB5 (*A. cryptum*-like), FKB6 (*Alb. acidocaldarius*-like) and FKA11 (*P. torridus*-like); these microbes may conduct heterotrophic lifestyle and obtain carbon through different kinds of transporters for DOC resources in the environment (Supplementary Table 7). The existence of such a distinct lifestyle is crucial for the AMD community, as the coexisting heterotrophic members could consume the DOCs, which are otherwise toxic to the autotrophs (Baker-Austin and Dopson, 2007). Multiple strategies for the utilization of nitrogen resources (for example, nitrogen, ammonium, nitrate, urea and so on) were identified in the AMD taxa, as reported in other AMD environments (Ram *et al.*, 2005; Bertin *et al.*, 2011; Méndez-García *et al.*, 2014). The nitrogen fixation *nif* genes were only expressed in the *At. ferrooxidans*-like FKB1 and *Leptospirillum* group III-like FKB4, which have been found to be the major or even the only nitrogen fixers in some AMD systems (Baker and Banfield, 2003; Goltsman *et al.*, 2009). Other taxa could obtain ammonium or nitrate alternatively, and FKB7 could also use urea as nitrogen resource. All these obtained nitrogen resources could be further used for glutamine and glutamate synthesis (Ertan, 1992). Unlike some subsurface mining environments where external nitrogen is limited (Tyson *et al.*, 2004; Bertin *et al.*, 2011; Méndez-García *et al.*, 2014), the Fankou AMD site is a ‘open' system and thus it is possible that part of the fixed nitrogen obtained by most of the taxa comes from external sources.

Many AMD microorganisms conduct energy conservation via the oxidation of reduced inorganic sulfur compounds (RISCs; Baker and Banfield, 2003; Johnson and Hallberg, 2003). In acidic mining environments, RISCs, including elemental sulfur (S^0^), thiosulfate (S_2_O_3_^2−^) and hydrogen sulfide (H_2_S), are generated via chemical oxidation of metal sulfides (Schippers and Sand, 1999). With these RISCs in AMD environment, AMD taxa could oxidize them with multiple enzymes, sulfite-quinone reductase can catalyze the oxidation of H_2_S to S^0^ and thiosulfate:quinone oxidoreductase catalyzes S_2_O_3_^2−^ to generate tetrathionate (S_4_O_6_^2−^), whereas tetrathionate hydrolase disproportionates S_4_O_6_^2−^ to S_2_O_3_^2−^, SO_4_^2−^ and S^0^ (Dopson and Johnson, 2012). So far, the Sox system has only been fully characterized in the model organism of *Paracoccus pantotrophus*, in which *SoxXA*, *SoxYZ*, *SoxB* and *SoxCD* together can mediate the oxidation of S_2_O_3_^2−^, SO_3_^2−^, S^0^ and H_2_S (Friedrich *et al.*, 2001). In contrast, the partial Sox system without *SoxCD* can only oxidize S_2_O_3_^2−^ to produce S^0−^(Hensen *et al.*, 2006). Our transcriptional analysis revealed the expression of multiple sulfur oxidation genes, including those coding sulfite-quinone reductase, thiosulfate:quinone oxidoreductase and tetrathionate hydrolase in FKB1 and FKB2, and the *Sox* system without *SoxCD* in FKB2. Partial *Sox* system has also been reported in the genus of *Acidithiobacillus*, for example, the *SoxYZ* in *At. ferrivorans* (Liljeqvist *et al.*, 2011) and *SoxAXBYZ* in *At. caldus* (Mangold *et al.*, 2011). With the activities of the sulfur-oxidizing enzymes in FKB1 and FKB2, SO_4_^2-^ and S^0^ may generate continuously, and the further oxidation of S^0^ to SO_4_^2−^ is key to the generation of acids because of release of protons in the reactions (Baker and Banfield, 2003). The resulting S^0^ could be oxidized to SO_3_^2−^ by several enzymes, such as the reverse function of dissimilatory sulfite reductase in green sulfur bacteria (Gregersen *et al.*, 2011), sulfur oxygenase reductase (SOR) in *At. caldus* (Mangold *et al.*, 2011) and *At. ferrivorans* (Liljeqvist *et al.*, 2011), or sulfur dioxygenase in *At. thiooxidans*, *A. acidophilum* and *A. cryptum* (Rohwerder and Sand, 2003). Although no dissimilatory sulfite reductase or SOR transcripts were detected, sulfur dioxygenase gene was expressed in FKB2 and FKB5. This indicated the key role of these two taxa in the acidification of sulfide minerals. Without their capabilities in further oxidation of S^0^, deposition of S^0^ would occur as previously reported in the extremely acidophilic sulfur-oxidizing biofilms (Johnson *et al.*, 2012) and thus the acid generation process may slow down. Finally, the obtained SO_3_^2−^ from sulfur dioxygenase could be oxidized to sulfate via adenylylsulfate by adenylylsulfate reductase and ATP sulfurylase by FKB1 and FKB5. Ferrous iron oxidation represents another energy conservation strategy for AMD microorganisms (Baker and Banfield, 2003), but the electron transporting pathways vary in different species (Bonnefoy and Holmes, 2012). In the Fankou community, several taxa may conduct iron oxidation as indicated by the expression of rusticyanin in FKB1, cyt_579_ and cyt_572_ in FKB3 and cyt_572_ in FKB4. The cyt_579_ was not detected via the functional annotation of the FKB4 genome, but this was likely due to the absence of reference sequences of this protein in the NBCI-nr database (Goltsman *et al.*, 2009). The FKB7 may also oxidize ferrous iron with the electron transporting pathway encompassing several putative proteins. These results suggest a putative role of FKB1, FKB3, FKB4 and FKB7 in iron oxidation in the AMD system, and these iron-oxidizers are further connected with the sulfur oxidizers by providing the effective oxidant ferric iron for the chemical oxidation of metal sulfides (see above).

Our analysis also identified multiple strategies used by the AMD taxa to adapt to the extreme conditions. For acid stress resistance, the taxa (FKB1, FKB2, FKB3, FKB4 and FKB5) may harbor an impermeable membrane via the biosynthesis of hopanoid as previously described in *Acidithiobacillus* and *Acidimicrobiaceae* spp. (Jones *et al.*, 2011), or use Na^+^/H^+^ antiporters (all eight active taxa) that were widely detected in AMD microorganisms (Tyson *et al.*, 2004). Other acid resistance strategies that consume ATP were also detected, including uptake of K^+^ in FKB1, FKB4, FKB5 and FKB6 and the use of proton sequestering molecules in FKB2, FKB3, FKB4, FKB6 and FKB7. Organic acids (for example, acetic, lactic, formic and propionic acid) were reported to be harmful to acidophiles because their protonated form could dissociate a proton in the cell (Ciaramella *et al.*, 2005), thus representing another acid stress. The *A. cryptum*-like FKB5 and *P. torridus*-like FKB11 may degrade organic acids as another stress resistance strategy by expressing the genes encoding propionyl-CoA synthase and acetyl-CoA synthetases (Baker-Austin and Dopson, 2007). Many heavy metal resistance transcripts were detected in all the bacterial taxa (COG3696, COG0841, COG1538 and COG1230), especially those for the efflux of Cd, Zn and Co, which were present at high levels in the AMD. The genes for Fe^2+^/Zn^2+^ uptake regulation proteins were also highly expressed in all bacterial taxa, likely indicating that uptake of the two meals is carefully balanced because of their high concentrations. Few of the above-mentioned heavy metals resistance genes were expressed in the *P. torridus*-like FKA11, implying that other novel strategies may be used for this archaeon. As previously described (Ram *et al.*, 2005), peroxiredoxin was widely used by all active AMD taxa to remit harm of oxidative stress, although other associated transcripts were also detected. In addition, the highly expressed chaperones genes in all active taxa may indicate a common mechanism for repairing DNA and protein damage because of the harsh conditions.

## Concluding remarks

We have attempted a novel pipeline to our short-read metagenomic and metatranscriptomic data sets, and recovered well-curated microbial genomes from both dominant and rare taxa in an extreme, low-diversity AMD system. In particular, we obtained the first genome of a naturally occurring *Ferrovum* population and reconstructed the metabolic pathways of these lesser known but ubiquitous acidophilic microorganisms. The identification and high expression of putative genes involved in iron oxidation indicated its key role in re-generating ferric iron, the primary sulfide oxidant in acidic mining environments. Our transcriptional analysis revealed multiple strategies for resource acquisition (carbon and nitrogen) and energy generation adopted by the AMD taxa through expressing both shared and taxon-specific genes. To survive in such an extreme habitat, these microbes have evolved various mechanisms to tolerate the low pH, high heavy metal concentrations and oxidative stress. Our results provide evidence that the coexistence of species is partly due to the facts that functional partitioning and rare members (for example, the N-fixing FKB1 and FKB4, and the S^0^ oxidizing FKB2 and FKB5) may play important ecological roles in the community. Our study highlights the power of the ‘divide and conquer' strategy for the assembly of genomes for both dominant and rare taxa from high-throughput sequencing data. With this strategy, the genomic and transcriptomic information of individual species in microbial communities along with environmental gradients or sampled at different time points could be captured, and subsequent comparative taxa transcriptional analyses may lead to a mechanistic understanding of the diverse responses of different taxa to environmental change.

## Figures and Tables

**Table 1:** Physical and chemical characteristics of the AMD sample

*pH*	*DO*	*TOC*	*Fe*^*2*+^	*Fe*^*3*+^	*SO*_*4*_^*2−*^	*Pb*	*Zn*	*Cu*	*Co*	*Cd*	*Cr*
2.6	6.0	18.5	6.2	193	474	1.5	186	2.3	4.5	4.5	2.2

Abbreviations: AMD, acid mine drainage; DO, dissolved oxygen; TOC, total organic carbon.

Concentrations are given in mg l^−1^ except for pH.

**Table 2:** Information of the 11 draft genomes assembled from metagenomic and metatranscriptomic data of the AMD community

*Draft genome*	*Closely related organism*	*Total bases*	*Number of*	*GC content*	*Completeness*	*16 S rDNA*
		*(bp)*	*contigs*	*(%)*	*(%)*^d^	*sequence*
FKB1	*Acidithiobacillus ferrooxidans*^a^	2 453 291	364	58.0	80.9	No
FKB2	*Acidithiobacillus thiooxidans*^a^	2 643 526	330	53.2	85.5	No
FKB3	*Leptospirillum rubarum* (group II)^a^	2 067 509	295	57.7	77.6	No
FKB4	*Leptospirillum ferrodiazotrophum* (group III)^a^	2 593 804	235	59.0	91.8	No
FKB5	*Acidiphilium cryptum*^a^^,^^c^	3 468 182	361	66.7	91.3	Yes
FKB6	*Alicyclobacillus acidocaldarius*^a^^,^^b^	2 449 390	286	45.5	75.7	No
FKB7	*Ferrovum* spp.^c^	2 983 188	276	40.1	79.0	Yes
FKA8	*Candidatus Micrarchaeum acidiphilum* ARMAN-2^a^^,^^c^	1 266 728	179	43.5	84.9	Yes
FKA9	*Candidatus Parvarchaeum acidiphilum* ARMAN-4^a^^,^^c^	1 223 344	203	35.9	79.1	Yes
FKA10	*Candidatus Parvarchaeum acidophilus* ARMAN-5^a^	1 041 772	193	39.9	74.1	No
FKA11	*Picrophilus torridus*^a^	1 113 980	213	40.9	53.2	No

a The taxonomic information was achieved by comparing contigs against NCBI-nr database using BLASTx.

b The nearest neighbor in maximum-likelihood phylogenies constructed with RAxML (v7.2.7) by combining 31 universal marker genes was identified as the closest organism.

c The taxonomic information was obtained by comparing 16 S rDNA sequence against NCBI-nt database using BLASTn.

d The completeness of each genome was estimated by the ratio of core genes observed in each genome and the corresponding pan-genome (see Supplementary Methods for details).

**Table 3:**
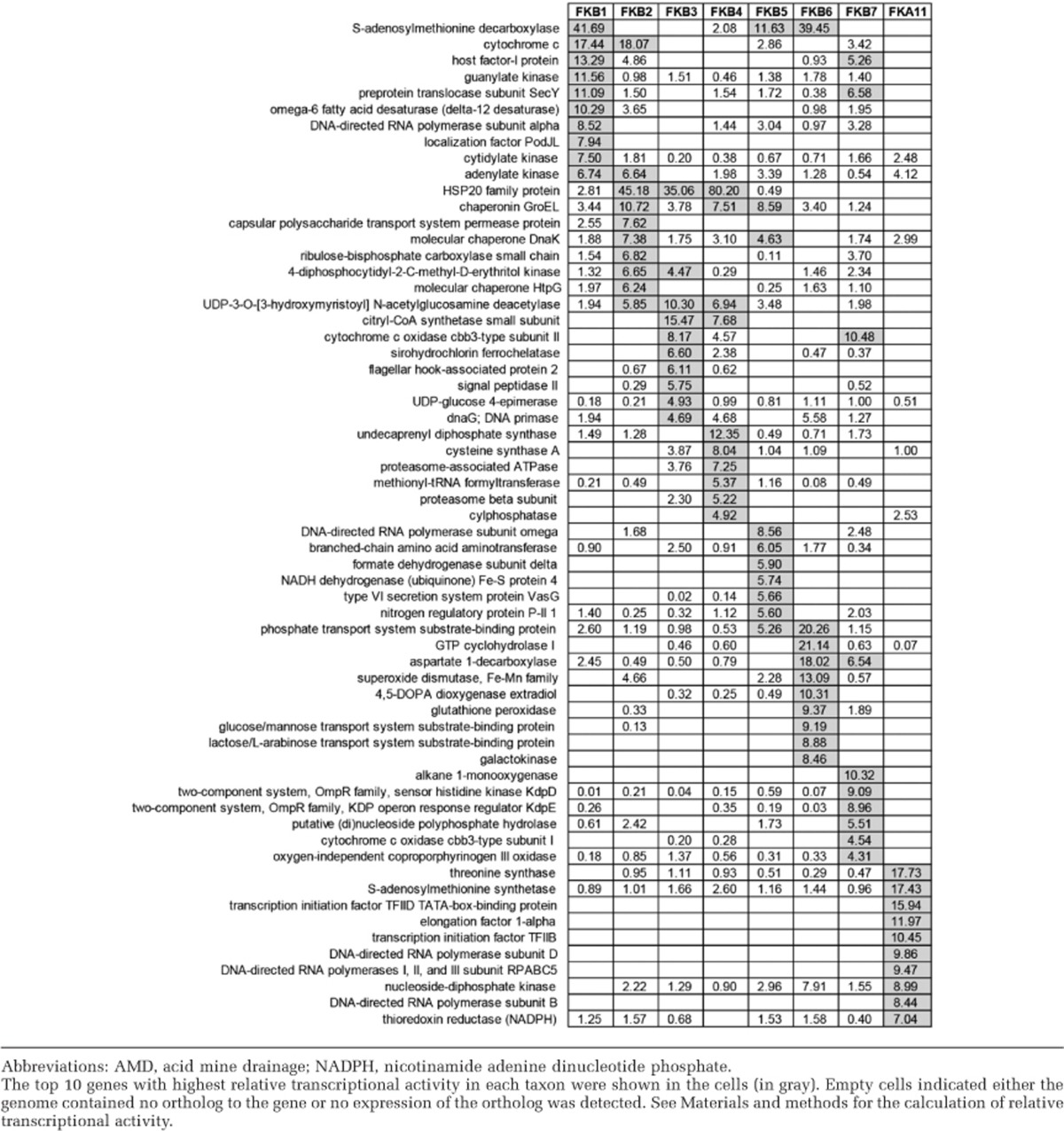
Top 10 highest expressed genes in the eight transcriptional active AMD taxa
