# Supplementary material for: Ecological roles of dominant and rare prokaryotes in acid mine drainage revealed by metagenomics and metatranscriptomics
Source: ISME J. 2014 Nov 7;9(6):1280–94. doi: 10.1038/ismej.2014.212 (PMC4438317; doi:10.1038/ismej.2014.212)
Supplement: Supplementary Information [file ismej2014212x1.pdf]

Supplementary Information

Supplementary methods, Figures S1-S11, Tables S1-S8

for

Ecological roles of dominant and rare prokaryotes in acid mine drainage revealed by metagenomics and metatranscriptomics

Zheng-shuang Hua[1,3], Yu-jiao Han[1,3], Lin-xing Chen[1,3], Jun Liu[1], Min Hu[1], Sheng-jin Li[1], Jia-liang Kuang[1], Patrick SG Chain[2], Li-nan Huang[1], Wen-sheng Shu[1]

[1]*State Key Laboratory of Biocontrol, Key Laboratory of Biodiversity Dynamics and Conservation of Guangdong Higher Education Institutes, College of Ecology and Evolution, Sun Yat-sen University, Guangzhou 510275, PR China*

[2]*Metagenomics Applications Team, Genome Science Group, Los Alamos National Laboratory, Los Alamos, NM, USA*

Correspondence: Wen-sheng Shu, College of Ecology and Evolution, Sun Yat-Sen University, Guangzhou 510275, PR China.

Tel.: +86 20 39332933; Fax: +86 20 39332944; E-mail: shuws@mail.sysu.edu.cn

or Li-nan Huang, College of Ecology and Evolution, Sun Yat-Sen University, Guangzhou 510275, PR China.

Tel.: +86 20 39332935; Fax: +86 20 39332944; E-mail: eseshln@mail.sysu.edu.cn

[3] These authors contributed equally to this work.

Supplementary methods

Sampling procedure

Acid mine drainage (AMD) samples were collected as previously described (Frias-Lopez *et al*., 2008). Briefly, a 5 L AMD sample was prefiltered through a 1.6-μm GF/A filter (149 mm diameter; Whatman) to remove particles or impurities, and further filtered through a 0.22-μm PES filter (149 mm diameter, PALL) to capture cells. The filters with cells were immediately flash frozen in liquid nitrogen to keep the integrity of the nucleic acids. A total of 10 filters were retained for subsequent experiments. Another four 5 L replicate AMD samples were collected for physicochemical analysis.

Physicochemical analysis

The physicochemical characteristics of the AMD samples were determined as previously described (Kuang *et al*., 2013). Briefly, temperature, pH and dissolved oxygen (DO) were measured on site using electrodes. The concentrations of ferrous iron (Fe^{2+}) and ferric iron (Fe^{3+}) were determined by ultraviolet colorimetric assays with 1,10-phenanthroline at 530 nm (Hill *et al*., 1978). The concentration of sulfate (SO_4^{2-}) was determined using a $BaSO_4$-based turbidimetric method (Chesnin and Yien, 1951). Heavy metals (including total Fe, Cd, Cr, Cu, Mn, Pb, Zn) were measured using inductively coupled plasma optical emission spectrometry (ICP-OES; Optima 2100DV; Perkin-Elmer, Massachusetts, USA).

DNA and RNA extraction, rRNA depletion, amplification and complementary DNA (cDNA) synthesis *and metagenomic and metatranscriptomic sequencing*

The genomic DNA was extracted from the microbial cells collected on filters as described previously (Fuhrman *et al*., 1988) and purified using QIAamp Mini spin columns (Qiagen, Hilden, Germany). The quality and quantity of the genomic DNA was estimated using agarose gel electrophoresis and a NanoDrop ND-1000 spectrophotometer (NanoDrop Technologies). Total RNA was extracted from filters using a modified mirVana miRNA Isolation kit (Ambion) protocol, as described previously (Shi *et al*., 2009). Briefly, tubes with filters were thawed on ice, added to lysis/binding buffer (Ambion), and vortexed to lyse the attached cells. Total RNA was then extracted from the lysate according to the manufacturer's protocol, and purified using the RNeasy MinElute Cleanup kit (Qiagen, Valencia, CA, USA). Subtractive hybridization with sample-specific biotinylated rRNA probes was used to remove bacterial 16S and 23S rRNA gene sequences from the total RNA samples. The probe synthesis reaction was modified from the DeLong *et al*. (1999) *in situ* hybridization method, combined

with the subtractive hybridization protocol of Su and Sordillo (1998). Ribonucleotide probes targeting bacterial 16S and 23S rRNA gene sequences were generated from community DNA samples collected in tandem with the total RNA samples. Templates for probe generation were first prepared by PCR using universal primers flanking the nearly full length 16S gene and ~85% of the 23S gene. The reverse primer was modified to contain the T7 RNA polymerase promoter sequence. PCRs (50 μl each) included 100 ng of template DNA, 1 μl of Herculase II Fusion DNA Polymerase (Stratagene, La Jolla, CA, USA), 1× Herculase reaction buffer, 10mM dNTP and 10 μM each of the forward and reverse primers. Reaction conditions were as follows: 2 min at 92 ℃; 35 cycles of 20 s at 95 ℃, 20 s at 39 ℃ (23S reactions) or 55 ℃ (16S reactions), 75 s (16S) or 90 s (23S) at 72 ℃; 3 min at 72 ℃. PCR products were purified via the QIAquick PCR purification kit (Qiagen). Biotinylated antisense rRNA probes were generated using *in vitro* transcription (IVT) with T7 RNA polymerase and the T7 promoter-containing 16S and 23S amplicons as templates. IVT was conducted using the MEGA script High Yield Transcription kit (Ambion), with the following modifications. Probes for 16S and 23S rRNA were generated separately in 2 μl reactions, each containing: 1× buffer, T7 RNA polymerase, SUPERase-In RNase inhibitor (10 U), ATP (7.5 mM), GTP (7.5 mM), CTP (5.625 mM), UTP (5.625 mM), biotin-11-CTP (1.875 mM, Roche), biotin- 16-UTP (1.875 mM, Roche) and 16S/23S DNA template (250–500 ng). Reactions were run at 37 ℃ for 4–5h, then DNAse digested with TURBO DNAse (Ambion) for 15 min at 37 ℃. Products were purified using the MEGAclear kit (Ambion). Biotinylated rRNA probes were hybridized to complementary rRNA molecules in the total RNA sample. The hybridization reactions (50 μl) contained formamide (20%), 1× SSC buffer (0.15M sodium chloride, 0.015M sodium citrate), SUPERase. RNase inhibitor (20 U), template RNA, and equal amounts of 16S and 23S rRNA probes (final template-to-probe ratio of 1:2) were denatured at 70 ℃ for 5min and incubated at room temperature (RT) for 3 min. Biotinylated double-stranded rRNA was then removed from the sample by hybridization (10min at RT) to streptavidin-coated magnetic beads (New England Biolabs, Ipswich, MA, USA; 50 μl aliquot, washed 3× in 1× SSC), followed by separation on a magnetic rack (2 min) and removal of the rRNA subtracted supernatant. An additional 50 μl 1× SSC was applied to the beads, separated as above, and pooled with the original supernatant. The pooled products were purified using the RNeasy MinElute Cleanup kit (Qiagen). The DNA and cDNA samples were sent to Macrogen Inc. (Seoul, Korea) for Illumina Hiseq 2000 paired-end sequencing.

16S rRNA library construction, Sanger sequencing and analysis

Bacterial and archaeal 16S rRNA genes were amplified by PCR, with the bacterial specific primers: 27F/1492R

and the archaeal specific primers: 21F/958R. The PCR reactions were conducted in triplicate as described previously (Fierer *et al.*, 2008). Clone libraries were constructed with the SureClone Ligation Kit (Amerham-Pharmacia) following the manufacturer's instructions. After gel-purification using E.N.Z.A. Gel Extraction Kit (Omega Bio-Tek, Inc.), PCR products were ligated into the pCR®2.1 vector with TA Cloning® Kit (Invitrogen, Inc.). The ligation mixtures were then transformed into *Escherichia coli* competent DH5α cells (Takara Biotechnology (Dalian) Co., Ltd.). Cells were grown in Luria-Bertani agar medium with Amp (100 mg/ml), X-Gal (20 mg/ml) and IPTG (24 mg/ml) at 37 ℃ for 16h. 40 white clones were sampled for each of the two libraries. All clones were sent to the Beijing Genomics Institute-Shenzhen (BGI, Shenzhen, China) for paired-end sequencing using the Sanger method. After quality trimming and vector removal using the Ribosomal Database Project (RDP) site, all near-full-length 16s rRNA gene sequences were clustered into OTUs (operational taxonomic units) at 97% similarity using Mothur (Schloss *et al.*, 2009). Taxonomic assignment of the OTU representative sequences was conducted via the RDP Classifier.

454 Pyrosequencing of bacterial and archaeal 16S rRNA genes and analysis

Barcoded pyrosequencing with the 515F/806R primer set (Caporaso *et al.*, 2011) targeting the V4 region of bacterial and archaeal 16S rRNA genes was conducted to investigate the microbial community structure and composition of the Fankou AMD sample. PCR reactions (30 μL) contained 0.75 units Ex Taq DNA polymerase (TaKaRa, Dalian, China), 1× Ex Taq loading buffer (TaKaRa, Dalian, China), 0.2 mM dNTP mix (TaKaRa, Dalian, China), 0.2 μM of each primer and about 100 ng of template DNA. PCR amplification was conducted as follows: initial denaturation at 95℃ for 3 min; 35 cycles of denaturation at 94℃ for 30 s, primer annealing at 50℃ for 1 min, extension at 72℃ for 1 min; a final extension of 10 min at 72℃. The PCR reaction was conducted in triplicate and the products were pooled to mitigate PCR amplification biases. Following gel purification using the QIAquick Gel Extraction Kit (Qiagen, Chatsworth, CA), the purified products were sent to Macrogen Inc. (Seoul, Korea) for pyrosequencing on a Roche 454 GS FLX Titanium pyrosequencer. Raw data were processed using both Mothur (version 1.29, Schloss *et al.*, 2009) and QIIME (Caporaso *et al.*, 2010). The data was denoised during the transformation of the flowgram to DNA sequences. Chimeric sequences were identified and removed. Sequences were then clustered into OTUs at the 97% similarity level, and taxonomic assignments were performed using the RDP Classifier (Wang *et al.*, 2007).

Bioinformatic analysis of metagenomic and metatranscriptomic datasets

Quality control of raw reads. Both raw metagenomic and metatranscriptomic reads generated by Illumina Hiseq2000 sequencer can contain artificial reads caused by adapter and contamination during library construction. Therefore, five steps were performed to obtain a clean read dataset with high quality. (1) Eliminate reads caused by adapter contamination; (2) Delete duplicated reads generated by PCR amplification specific to long insert-sizes; (3) Remove reads with a significant excess of "N" ($\geq$ 10% of the read). (4) Filter out low complexity reads using the SGA preprocess (Simpson *et al.*, 2012) program. (5) Trim the reads with Q score less than 15 at the 3' ends.

16S rRNA gene bearing reads identification. The quality metagenomic reads were then mapped to the OTUs representative sequences of the 454 pyrosequencing analysis via BLASTn, those reads with a e-value $\leq 10^{-5}$ were identified as 16S rRNA gene bearing reads. With the taxonomic assignments of the OTUs, we thus calculated the microbial composition based on these 16S rRNA gene bearing reads (Supplementary Figure 3).

Assembly. Because of lack of sufficient memory during Velvet assembly (version 1.1.06; Zerbino and Birney, 2008) despite our 512 GB RAM server, khmer (version 0.3; Pell *et al.*, 2012) was required. This step uses a simple probabilistic representation for storing de Brujin graphs in memory based on Bloom filters. The reads were partitioned into two components based on their k-mer (k=31 in our case) depth (see materials and methods), which can help make it possible to assemble genomes from both high- and low-abundance populations from the deep sequenced metagenomic and metatranscriptomic data (Hess *et al.*, 2011). To get longer contiguous sequences, we merged results produced using multiple k-mer assemblies (Mason *et al.*, 2012) and the metatranscriptome assembly by Trinity (Grabherr, 2011) which can benefit transcriptional analysis.

Contig coverage calculation. To compute the coverage information of each contig, reads were mapped to the combined draft genomes using Bowtie (Langmead *et al.*, 2009). For each read, only the best alignment was kept, allowing for up to 3 errors in the first 20 bases, detailed information about the parameters as follows: -k 1 -e 180 -n 3 -l 20 --best --tryhard --chunkmbs 128. The SAM format output was transformed into BED format using samtools (Li *et al.*, 2009) and bedtools (Quinlan and Hall, 2010). We used customed perl scripts to count the total bases mapped to one contig and divided it by contig length to calculate the coverage information.

Genome binning. Using supervised classifiers, including BLAST against the NCBI-nr (or nt) database or

phymmBL against thousands of complete/draft genomes, most contigs could be assigned to particular community members. For contigs ≥ 3000bp, we compared them against the NCBI-nr protein database using BLASTx (e-value ≤ 10^{-5}), and also against >1900 complete/draft genomes and nine AMD draft genomes using phymmBL. It is well recognized that supervised classification approaches introduce biases due to the unequal representation of microbial taxonomic groups in the public databases (Huson *et al*., 2007). Thus, unsupervised approaches were also employed. First, tetranucleotide (136 kinds) frequency (TNF) of the contigs with length ≥ 3000bp was calculated using in-house perl script. TNF based Hierarchical Agglomerative Clustering was then performed on these contigs. Also, Non-metric Multidimensional scaling (NMDS) analysis was conducted based on the contigs' TNF distance matrix. And the most appropriate number of bins the contigs should be grouped into (11 in our case) was determined based on the clustering and NMDS results. In each bin, contigs with a phylogenetic assignment (order level; based on BLASTx and phymmBL results) different from the majority of others were removed, and contigs with coverage at an inconsistent level with mean and standard deviation of the bin were also removed (Hess *et al*., 2011). All removed contigs were inspected and manually merged into the most possible bins or deleted based on their taxonomic information. Subsequently, assembly errors such as inappropriate linkage between contigs within each bin were detected by BLASTn, and most were found to be due to repeat regions. For this reason, the contigs in each bin were shredded into 500 bp overlapped fake "reads" using an in-house perl script and reassembled using phrap (http://bozeman.mbt.washington.edu/phrap.docs/phrap.html). All these procedures provided the final 11 final draft genomes for further analysis.

ESOM confirmation of genome binning. To evaluate the efficiency of genome binning results, ESOM analysis of genomic sequences based on tetranucleotide frequency (5000 bp window size; contigs ≥ 3000 bp) was conducted. The raw tetranucleotide frequencies were transformed with the "Robust ZT" in the Databionics ESOM tools (Ultsch and Moercen, 2005). Maps were toroidal (borderless) with Euclidean grid distance and dimensions of 80 × 150. Training was conducted using the K-Batch algorithm (k = 0.15 in percent) for 20 training epochs. The standard best match search method was used with local best match search radius of 8. Other training parameters were as follows: Gaussian weight initialization method; Euclidean data space distance function; starting value for radius of 24 and final value for radius of 1; starting value for learning rate of 0.5 with linear cooling to 0.1; Gaussian kernel function.

Genome completeness. To estimate the completeness of the genome bins (see Table 2), we first determined their taxonomic assignment using the phylogenetic tree constructed with the concentrated proteins sequences of 31 universal and rarely transferred horizontally marker genes (see below). For each genome bin, finished near neighbor genome sequences were manually selected and downloaded from the IMG database and used to determine the pan-genome at the order level (Supplementary Table 8). COG genes shared by selected genomes were defined as the core gene set of the pan-genome. Then we estimated the genome completeness by computing the ratio of core genes observed in each genome bin and corresponding pan-genome.

Genome accuracy. To estimate the accuracy of each genome bin, large-scale nucleotide alignments were performed using MUMmer (Delcher *et al.*, 2003) between the contigs of our draft genomes and their nearest neighbors extracted from the NCBI RefSeq database. First, all the maximal unique matches were obtained through the nucmer program. Non credible matches were filtered out with the delta-filter program based on default parameters. The alignments were visualized using dot plots (see Supplementary Figure 5).

Gene abundance computation. To calculate the abundance of each predicted protein-coding gene in the metagenomic and metatranscriptomic datasets, we mapped our metagenomic and metatranscriptomic reads separately to the combined 11 draft genomes reference using Bowtie. For each read, only the best alignment was kept, allowing for up to 3 errors in the first 20 bases, detailed information about the parameters as follows: -k 1 -e 180 -n 3 -l 20 --best --tryhard --chunkmbs 128. For a given predicted protein-coding gene, the number of reads (with 100% of their length aligned) were used to calculate the abundance of the gene in (using DNA or cDNA).

Phylogenetic tree reconstruction. To access the phylogenetic information of the assembled genomes, 658 fully sequenced prokaryotic genomes were selected manually to build a phylogenetic tree based on the concentrated proteins sequences of 31 universal and rarely transferred horizontally marker genes (Ciccarelli *et al.*, 2006). For each marker, the corresponding family of clusters of orthologous groups (COGs) from the STRING (version 9.0, Szklarczyk *et al.*, 2011) database was identified. Separate multiple sequence alignments (MSAs) of each COG were built for *Archaea* and *Bacteria* domains using MUSCLE (Edgar, 2004) respectively (iterate 100 times). For each domain, we concentrated the 31 MSAs and eliminated poorly aligned regions using Gblocks (options: -t=p -b3=8 -b4=2 -b5=h, Castresana, 2000). To reconstruct a phylogenetic tree integrating *Archaea* and *Bacteria* domains, the Gblocks cut MSAs from *Archaea* and *Bacteria* domains were aligned using ClustalW (Thompson

et al., 1994). The phylogenetic tree was constructed using RAxML (options: -m PROTGAMMAIWAGF -T 40 -c 8 -e 0.001, Stamatakis, 2006).

References

Caporaso JG, Kuczynski J, Stombaugh J, Bittinger K, Bushman FD, Costello EK *et al*. (2010). QIIME allows analysis of high-throughput community sequencing data. *Nat Methods* **7**: 335-336.

Ciccarelli FD, Doerks T, Von Mering C, Creevey CJ, Snel B, Bork P. (2006). Toward automatic reconstruction of a highly resolved tree of life. *Science* **311**: 1283-1287.

Chesnin L, Yien CH. (1951). Turbidimetric determination of available sulphates. *Proc Soil Sci Soc Am* **15**: 149-151.

Delcher AL, Salzberg SL, Phillippy AM. (2003). Using MUMmer to identify similar regions in large sequence sets. *Curr Protoc Bioinformatics* 10-3.

Edgar RC. (2004). MUSCLE: multiple sequence alignment with high accuracy and high throughput. *Nucleic Acids Res* **32**: 1792-1797.

Fierer, N., Hamady, M., Lauber, C. L., & Knight, R. (2008). The influence of sex, handedness, and washing on the diversity of hand surface bacteria. *Proc Natl Acad Sci USA* **105**: 17994-17999.

Frias-Lopez J, Shi Y, Tyson GW, Coleman ML, Schuster SC, Chisholm SW *et al*. (2008). Microbial community gene expression in ocean surface waters. *Proc Natl Acad Sci USA* **105**: 3805-3810.

Fuhrman SA. (1988). Appropriate laboratory testing in the screening and work-up of Cushing's syndrome. *Am J Clin Pathol* **90**: 345.

Hess M, Sczyrba A, Egan R, Kim TW, Chokhawala H, Schroth G *et al*. (2011). Metagenomic discovery of biomass-degrading genes and genomes from cow rumen. *Science* **331**: 463-467.

Hill AG, Bishop E, Coles LE, McLaughlan EJ, Meddle DW, Pater MJ *et al*. (1978). Standardized general method for the determination of iron with 1,10-phenanthroline. *Analyst* **103**: 391-396.

Huson DH, Richter DC, Rausch C, Dezulian T, Franz M *et al*. (2007). Dendroscope: An interactive viewer for large phylogenetic trees. *BMC Bioinformatics* **8**: 460.

Grabherr MG, Haas BJ, Yassour M, Levin JZ, Thompson DA, Amit I *et al*. (2011). Full-length transcriptome assembly from RNA-Seq data without a reference genome. *Nat Biotechnol* **29**: 644-652.

Kuang JL, Huang LN, Chen LX, Hua ZS, Li SJ, Hu M *et al.* (2013). Contemporary environmental variation determines microbial diversity patterns in acid mine drainage. *ISME J* **7**:1038-1050.

Langmead B, Trapnell C, Pop M, Salzberg SL. (2009). Ultrafast and memory-efficient alignment of short DNA sequences to the human genome. *Genome Biol* **10**: R25.

Li H, Handsaker B, Wysoker A, Fennell T, Ruan J, Homer N *et al*. (2009). The sequence alignment/map format and SAMtools. *Bioinformatics* **25**: 2078-2079.

Mason OU, Hazen TC, Borglin S, Chain PS, Dubinsky EA, Fortney JL *et al*. (2012). Metagenome, metatranscriptome and single-cell sequencing reveal microbial response to Deepwater Horizon oil spill. *ISME J* **6**: 1715-1727.

Pell J, Hintze A, Canino-Koning R, Howe A, Tiedje JM, Brown CT. (2012). Scaling metagenome sequence assembly with probabilistic de Bruijn graphs. *Proc Natl Acad Sci USA* **109**: 13272-13277.

Quinlan AR, Hall IM. (2010). BEDTools: a flexible suite of utilities for comparing genomic features. *Bioinformatics* **26**: 841-842.

Saitou N, Nei M. (1987). The neighbor-joining method: a new method for reconstructing phylogenetic trees. *Mol Biol Evol* **4**: 406-425.

Schloss PD, Westcott SL, Ryabin T, Hall JR, Hartmann M, Hollister EB *et al*. (2009). Introducing mothur: open-source, platform-independent, community-supported software for describing and comparing microbial communities. *Appl Environ Microbiol* **75**: 7537-7541.

Shi Y, Tyson GW, DeLong EF. (2009). Metatranscriptomics reveals unique microbial small RNAs in the ocean's water column. *Nature* **459**: 266-269.

Simpson JT, Durbin R. (2012). Efficient de novo assembly of large genomes using compressed data structures. *Genome Res* **22**: 549-556.

Stamatakis A. (2006). RAxML-VI-HPC: maximum likelihood-based phylogenetic analyses with thousands of taxa and mixed models. *Bioinformatics* **22**: 2688-2690.

Szklarczyk D, Franceschini A, Kuhn M, Simonovic M, Roth A, Minguez P *et al*. (2011). The STRING database in 2011: functional interaction networks of proteins, globally integrated and scored. *Nucleic Acids Res* **39**: D561-D568.

Tamura K, Peterson D, Peterson N, Stecher G, Nei M, Kumar S. (2011). MEGA5: molecular evolutionary genetics analysis using maximum likelihood, evolutionary distance, and maximum parsimony methods. *Mol Biol Evol* **28**: 2731-2739.

Thompson JD, Higgins DG, Gibson TJ. (1994). CLUSTAL W: improving the sensitivity of progressive multiple sequence alignment through sequence weighting, position-specific gap penalties and weight matrix choice.

Nucleic Acids Res **22**: 4673-4680.

Ultsch A, Moerchen F. (2005). ESOM-Maps: tools for clustering, visualization, and classification with Emergent SOM. *Technical Report of the Department of Mathematics and Computer Science, University of Marburg, Germany* 46: 1–7.

Wang Q, Garrity GM, Tiedje JM, Cole JR. (2007). Naive Bayesian classifier for rapid assignment of rRNA sequences into the new bacterial taxonomy. *Appl Environ Microbiol* **73**: 5261-5267.

Zerbino DR, Birney E. (2008). Velvet: algorithms for de novo short read assembly using de Bruijn graphs. *Genome Res* **18**: 821-829.

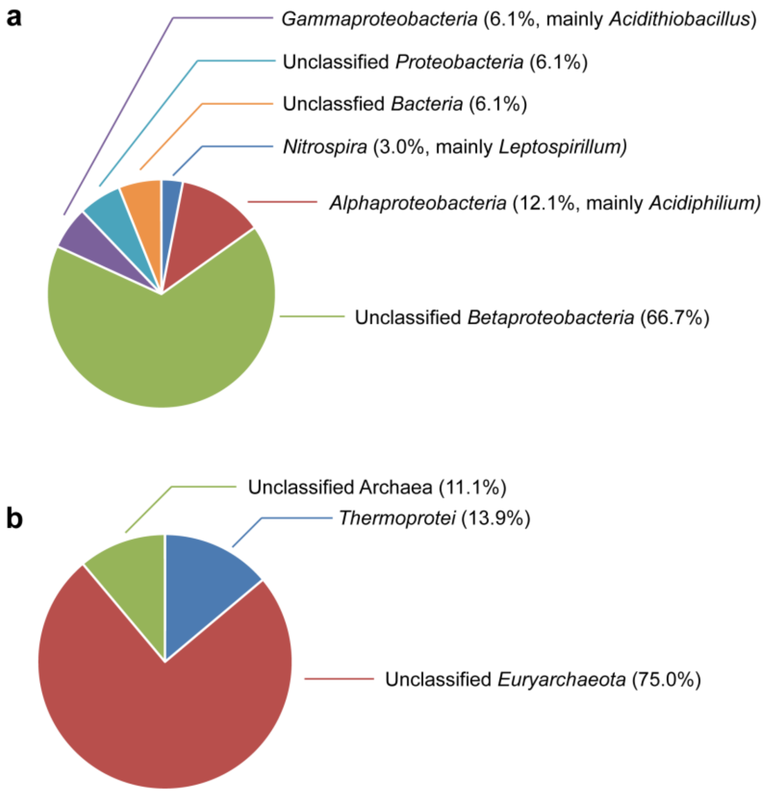

Figure S1 Taxonomic composition of Fankou AMD sample as revealed by 16S rRNA gene based clone library analysis. (a) Bacterial composition; (b) Archaeal composition.

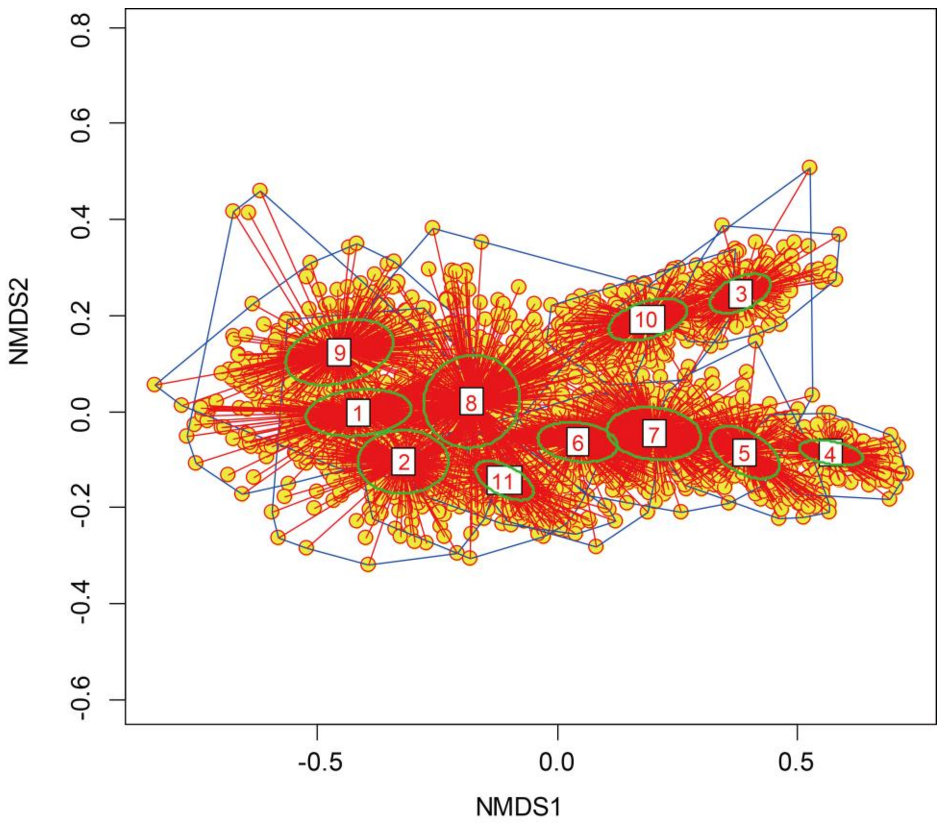

Figure S2 The ordination plot of non-metric Multidimensional scaling (NMDS) analysis (dimension = 2) conducted for all the contigs ≥ 3000 bp based on their TNF distance. In combination with clustering analysis results, 11 bins were deemed be appropriate for binning the contigs (see Supplementary methods)..

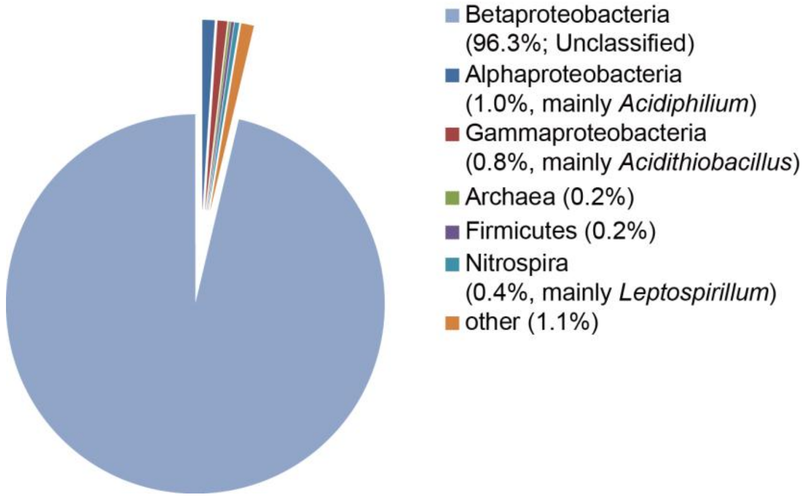

Figure S3 The taxonomic composition of the AMD system based on 16S rRNA gene bearing reads in the metagenomic dataset. This was done by mapping the quality metagenomic reads to the representative sequences of OTUs from 454 pyrosequencing analysis (see Supplementary methods for details).

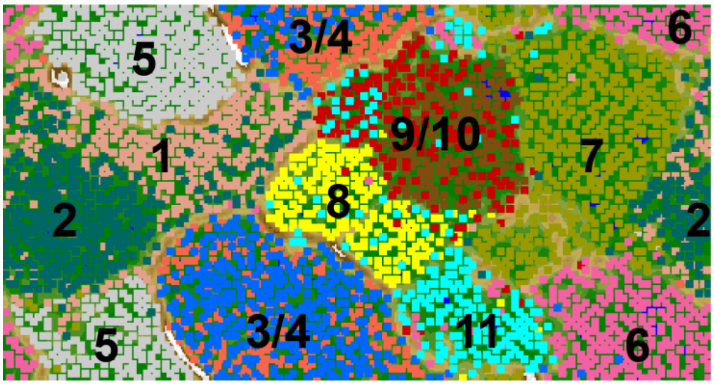

Figure S4 Genome bins reconstructed from the integrated metagenomics and metatranscriptomic data distinguished by ESOM. Clusters are: 1, FKB1 (*Acidithiobacillus ferrooxidans*-like); 2, FKB2 (*Acidithiobacillus thiooxidans*-like); 3, FKB3 (*Leptospirillum rubarum*-like); 4, FKB4 (*Leptospirillum ferrodiazotrophum*-like); 5, FKB5 (*Acidiphilium cryptum*-like); 6, FKB6 (*Alicyclobacillus acidocaldarius*-like); 7, FKB7 (*Ferrovum* spp.); 8, FKA8 (*Candidatus Micrarchaeum acidiphilum* ARMAN-2-like); 9, FKA9 (*Candidatus Parvarchaeum acidiphilum* ARMAN-4-like); 10, FKA10 (*Candidatus Parvarchaeum acidophilus* ARMAN-5-like); 11, FKA11 (*Picrophilus torridus*-like).

Figure S5 Maximum-likelihood phylogenetic tree based on a concatenation of 31 broadly conserved orthologs in 669 bacterial and archaeal genomes (containing 658 fully sequenced genomes and the assembled 11 draft genomes). Phyla are distinguished by the color of branch.

The Figure is provided as separate file (Supplementary Figure 5) for it is too large to integrate.

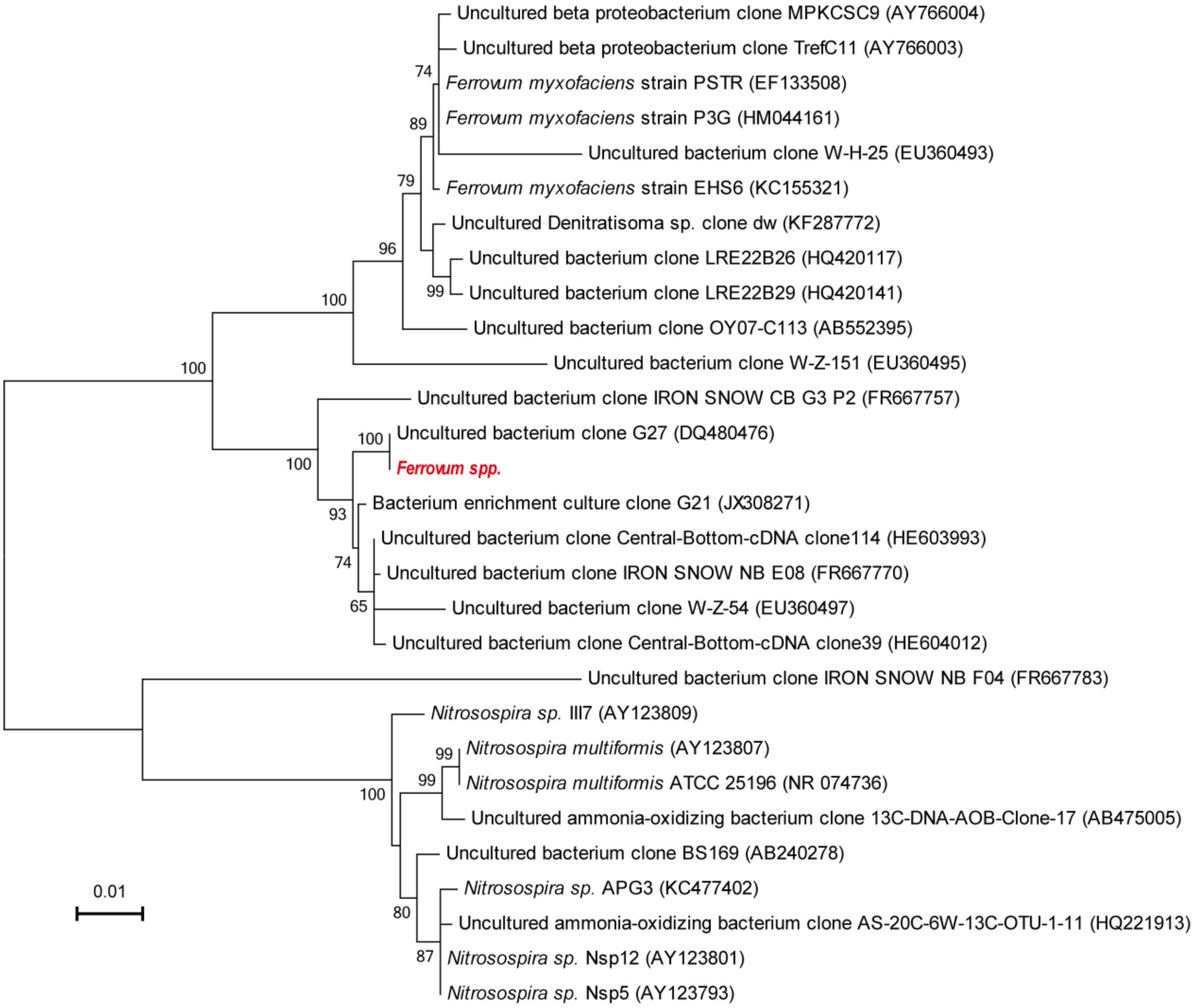

Figure S6 Phylogenetic tree (maximum likelihood, bootstrap = 1000) of *Ferrovum*-like FKB7 and selected related species based on 16S rRNA genes (>1400 bp in length). Bootstrap values (only bootstrap value > 50% is shown) are indicated at the nodes.

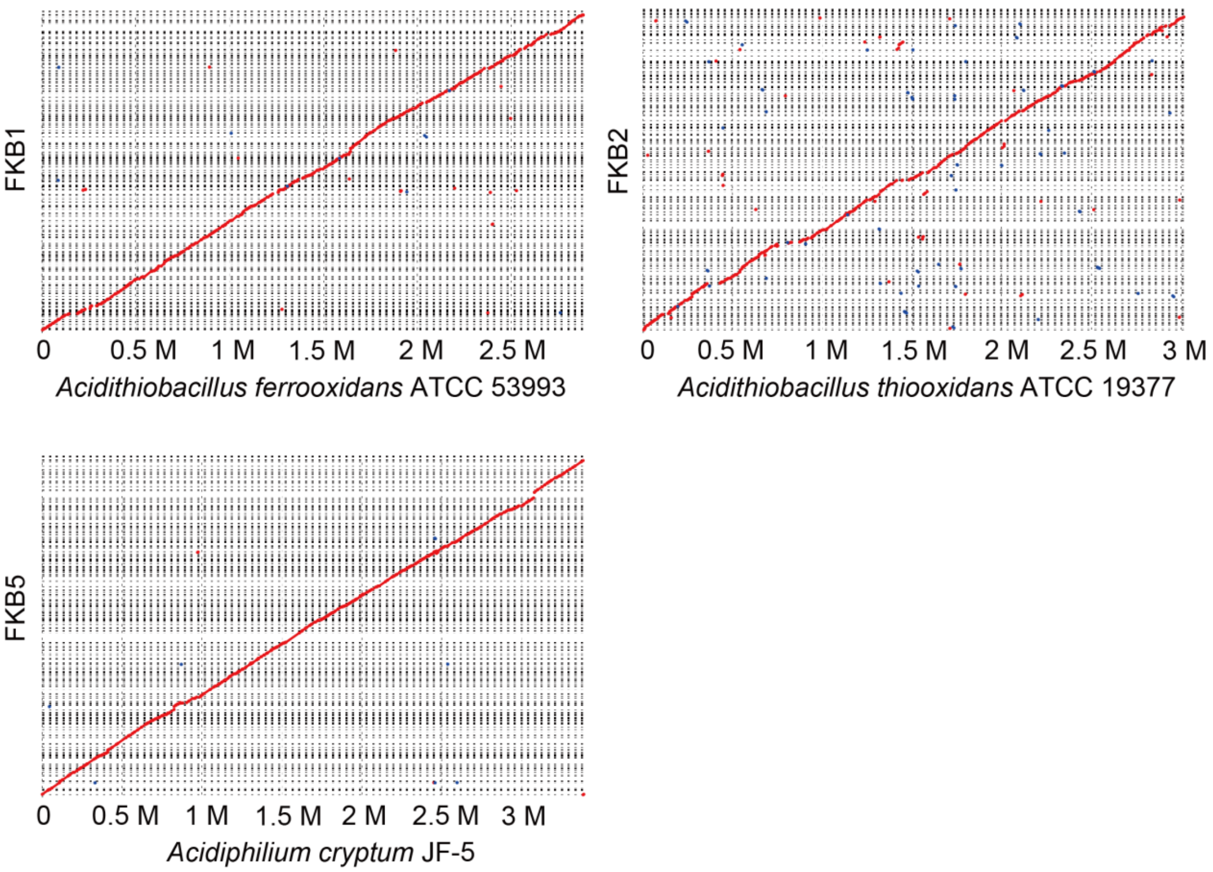

Figure S7 The alignments between draft genomes we assembled and the reference genomes (The accession numbers: *Acidithiobacillus ferrooxidans* ATCC 53993, NC_011206; *Acidithiobacillus thiooxidans* ATCC 19377, AFOH01). The sequences are ordered with respect to the reference genomes, forward maximal unique matchers (MUMs) of draft genome sequences against that of reference genome are plotted as red dots and reverse MUMs are plotted as blue ones. The plot was generated by MUMmer (Delcher AL, *et al.* 2003).

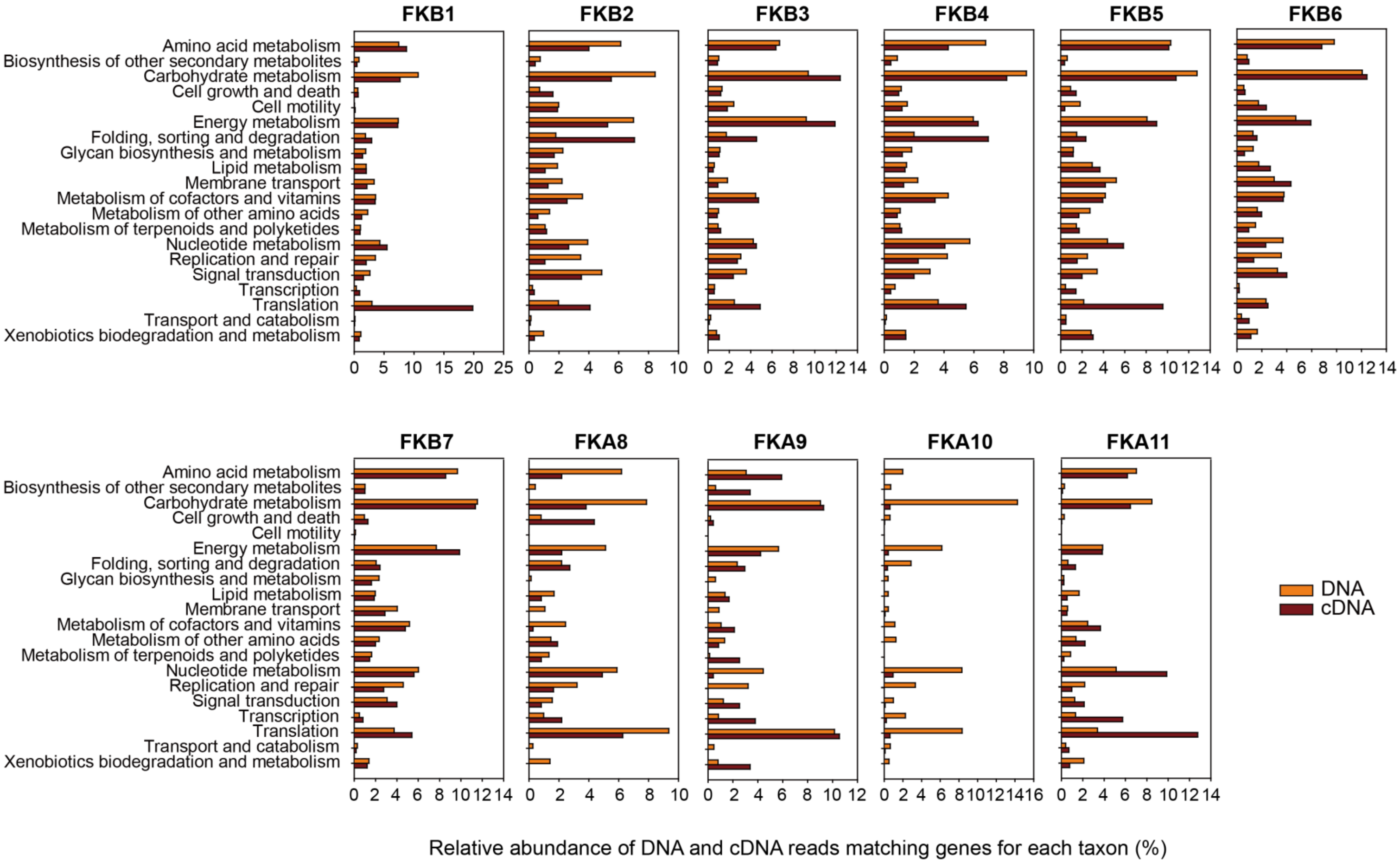

Figure S8 Relative abundance of DNA and cDNA reads assigned to genes of KEGG categories for the AMD taxa, which was calculated as the number of DNA (or cDNA) reads assigned to a KEGG category dividing by the total DNA (or cDNA) reads assigned to the taxon.

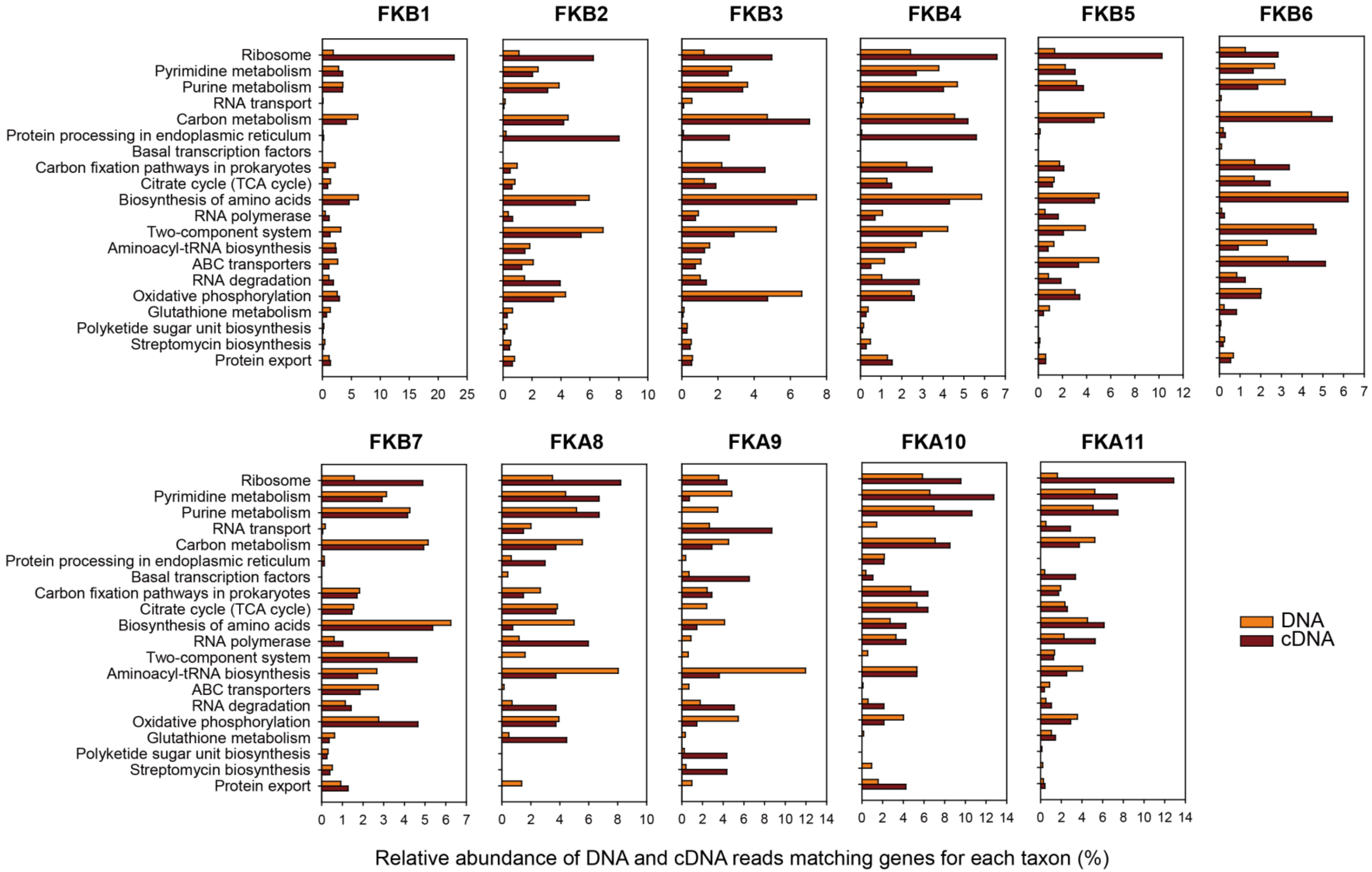

Figure S9 Relative abundance of DNA and cDNA reads assigned to genes of KEGG metabolic pathways for the AMD taxa, which was calculated as the number of DNA (or cDNA) reads assigned to a KEGG metabolic pathway dividing by the total DNA (or cDNA) reads assigned to the taxon. Only the top 20 most highly expressed KEGG pathways are shown.

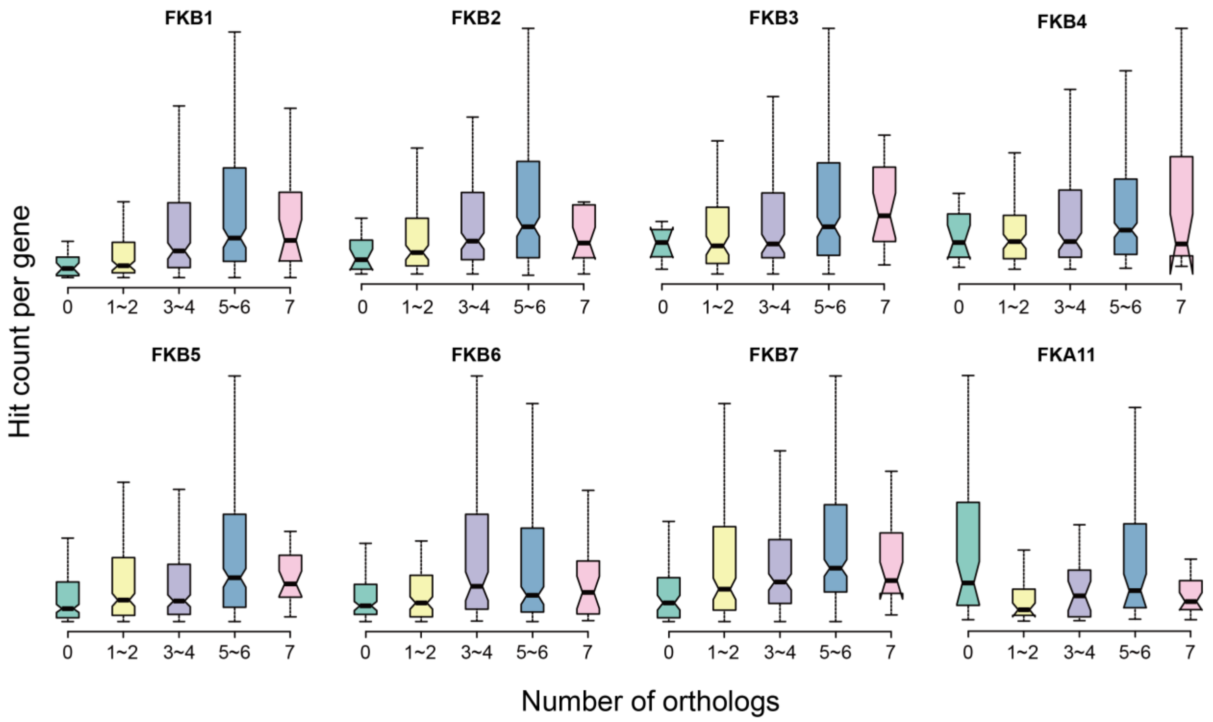

Figure S10 Expression level (hit count) as a function of ortholog number (representation in the other 7 draft genomes). Error bars denote 1.5 times the interquartile range. The magnitude of the y-axis varies for each genomes and is not shown for clarity.

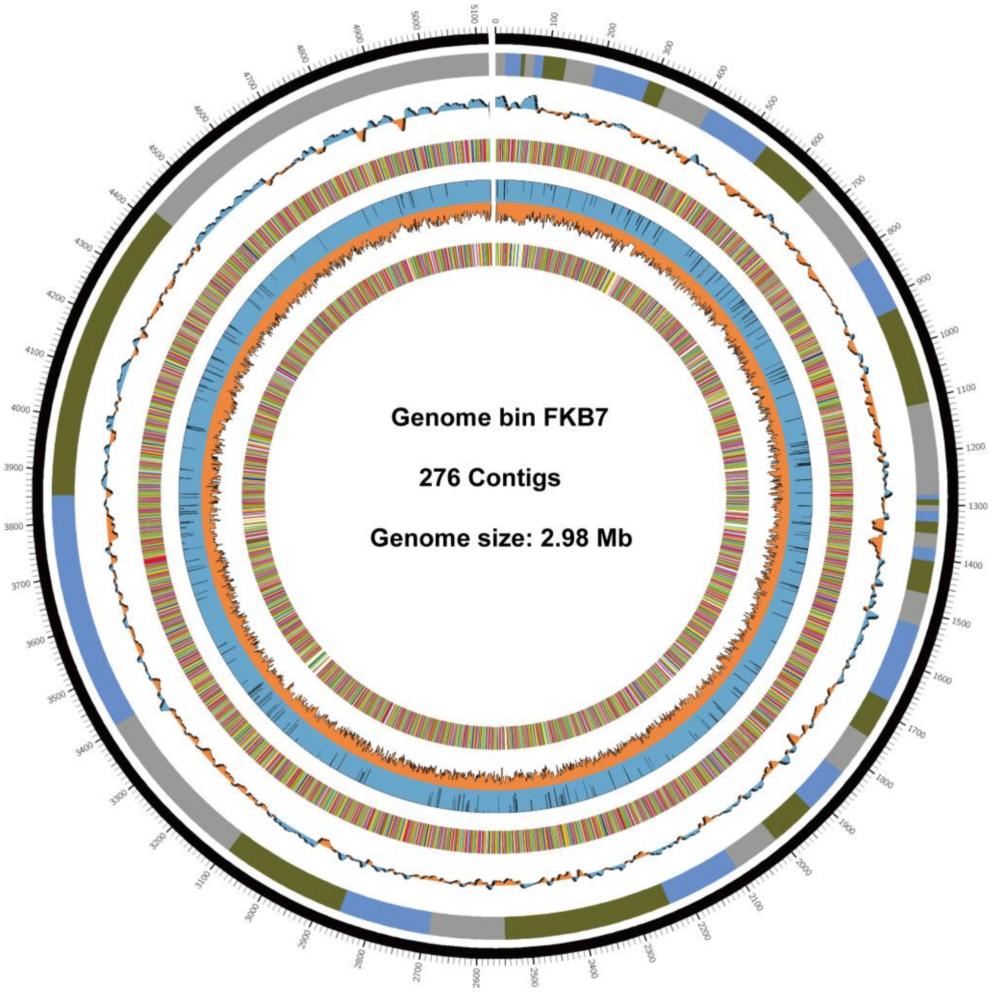

Figure S11 Circular representation of the draft genome of *Ferrovum*-like FKB7 reconstructed from metagenomic and metatranscriptomic datasets. Outermost circle, contigs within the draft genome (contigs were concatenated in random order); circle 2 shows the bin's guanine and cytosine content (GC%, blue > 39.9% and orange < 39.9% on average); circle3 shows all the open reading frames (ORFs) which are predicted using Meta-Genemark package (Zhu *et al.* 2010); circle 4, gene coverage (the natural logarithm of average coverage for each gene) in the metagenomic data is shown in outward orientation in blue and gene similarity compared to NCBI-nr database using BLASTX in the opposite direction in orange; innermost circle shows all the genes matching the COG database.

>afr:AFE_3152 cyc1; cytochrome c552 (A)
Length = 230

Score = 85.1 bits (209), Expect = 6e-23
Identities = 61/179 (34%), Positives = 86/179 (47%), Gaps = 1/179 (0%)
Frame = +1

```
Query  151   CMTCHGPTGWGTEAMGAPRLANLGYPYIVKQLTDLAEGRRVPQGAGAVMPYFASQLTPQE  330
             CM CHG TG  T     PRLA     Y+  QL    +  R  Q     M   A  L   +
Sbjct  63    CMVCHGMTGRDTLYPIVPRLAGQHKSYLEAQLKAYKDHSRADQNGEIYMWPVAQALDSAK  122

Query  331   RKDIAAYVNTLNTTPELSDIKEIQSSGQPVGVRYKGEILVKYGVEN-KVSACVSCHGYNG  507
                +A Y N      + S IK         GV+ +G+ +   GV N ++ AC+ CHG  G
Sbjct  123   ITALADYFNAQKPPMQSSGIKH-------AGVK-EGKAIFNQGVTNEQIPACMECHGSAG  174

Query  508   RGAPPMFPVIGQQKFTYLVNQLHNWRDGSRANDPLGMMRAIAKNLSDQDILDAATYLSS  684
             +GA P FP +  Q++ Y++ QL  +  G+R N    +M  IAKN++   + D A YLSS
Sbjct  175   QGAGP-FPRLAGQRYGYIIQQLTYFHNGTRVNT---LMNQIAKNITVAQMKDVAAYLSS  229
```

>afr:AFE_3153 cyc2; cytochrome c (A)
Length = 485

Score = 130 bits (327), Expect = 5e-36
Identities = 137/498 (27%), Positives = 207/498 (41%), Gaps = 33/498 (6%)
Frame = +1

```
Query  43    LALMGGVVLLSSLDANAIPVFARQTGFKCVACHVGGEYPQLTALGRYFKLTGYT------  204
             LA +GG+ L S   A A+P FARQTG+ C ACH    YPQLT +GR FKL G+T
Sbjct  17    LAAVGGMALSSG--AWALPSFARQTGWSCAACHT--SYPQLTPMGRMFKLLGFTTTNLQR  72

Query  205   ----QGDAKNIFGEMTTNLDRSPFSVWLQASKQWYANTQP-----NGINGTAPTSAFD-A  354
                 Q    N  G + + +  S FS++LQAS       Q      N   G +P +
Sbjct  73    QQKLQAKFGNSVGLLISRV--SQFSIFLQASATNVGGGQAVFGPGNSNAGASPNNNVQFP  130

Query  355   QTISLFTGGHLTDNLGAFIQWTAAKYDPLTTVSTGPSAPLNWTVSIDNSEVRLADHRVNS  534
             Q +SLF  G +T ++G+F+         LT    G  A        D+S V     ++ +
Sbjct  131   QQVSLFYAGEITPHIGSFLH--------LTYSGGGSGAGAGGFSFDDSSIVWTHPWKLGT  182

Query  535   SGDWIYGAYLNNRITMSDVWNTQENWTSDWIGYFNTGFNGVAGLAPQTQLQGGL-SQHNV  711
             +   + G  +NN  T  D+WNT    T DW   F +      G  PQ  ++    + + +
Sbjct  183   NNLLVTGVDVNNTPTAMDLWNT----TPDWQAPFFSSDYSSWGHVPQPFIESSAGAGYPL  238

Query  712   VGLGTY---LF--KDKTW-YGELGVY---RKVSNGPLSVFT----EGVDAASAPSYNNNL  852
              G+G Y   +F      W Y +  VY   +     P+  FT    +G  +  AP
Sbjct  239   AGVGVYGADIFGPNRANWLYADADVYTNGQGTQVNPVGGFTAAGPQGRLSGGAP------  292

Query  853   YTRFGYNKEWGPHEVFFGLHGLFGSGDPYGVNNLFSLANAQA-TWQDMGVDAQYQYILDP  1029
             Y R  Y  +WG      G  G++ S     +NN  + A     T+ D  +D Q Q++
Sbjct  293   YVRLAYQHDWGDWNWEVGTFGMWSSVYDNTINNTLNKAGGPIDTFDDYDLDTQLQWLDTN  352

Query  1030  HYFAAHFRLTHENMQNSAPLVGGVVANNANTLNEYWADMTYIYKAKYGAMLFFHGARGTN  1209
                    R    N Q        + +N++  LN +  + TY Y   YG    +    G+
Sbjct  353   DNNNVTIRAAWVNEQQQFGAGNVISSNSSGNLNFFNINATYWYHDHYGIQGGYRNVWGSA  412

Query  1210  DTTLYSSGV--NGSPDWDSIMPSVFWAPYQNIRIGVMETFYTRLGGTTSNLITNANNLPG  1383
             +  LY +    +GSPD  +      + P+ N R  +    Y +  G  S    ++NNL
Sbjct  413   NPGLYGTTYTNSGSPDTSNEWIEASYLPWWNTRFSLRYVVYNKFNGVGS---ASSNNLGY  469

Query  1384  SLSPHSFNTTMLYGSVIY  1437
               S  ++NT  L   + Y
Sbjct  470   GAS--AYNTLELLAWISY  485
```

>gi|1708556|sp|P50500.1|IRO_ACIFR RecName: Full=Iron oxidase; AltName: Full=Fe(II) oxidase; Flags: Precursor
Length = 90

Score = 39.3 bits (90), Expect = 2e-08
Identities = 27/82 (32%), Positives = 40/82 (48%)
Frame = +1

```
Query  40    MGSIALVAAPLVGFSTGASAKQNAAVRAALHFQNKPNGAKHCAVCMNFLPNKANLDHSGC  219
             +GS+A      VG +   S       +AA+ +Q+ P G  HC+VC  F+       HS C
Sbjct  21    VGSVATTTMMGVGVADAGSMP-----KAAVQYQDTPKGKDHCSVCAQFI-----APHS-C  69

Query  220   KLYPGDDEICQNCYCNGFVQKA  285
             K+  G+  I  N +C  FV K+
Sbjct  70    KVVAGN--ISPNGWCVAFVPKS  89
```

>gi|344199577|ref|YP_004783903.1| iron oxidase [Acidithiobacillus ferrivorans SS3]
Length = 90

Score = 35.4 bits (80), Expect = 6e-07
Identities = 24/89 (26%), Positives = 39/89 (43%)
Frame = +1

```
Query  19    SRRQLLKMGSIALVAAPLVGFSTGASAKQNAAVRAALHFQNKPNGAKHCAVCMNFLPNKA  198
             +RR  LK  ++ + A           A      +AA+ +Q+ P G   C++C  F+
Sbjct  9     TRRDALKNIAVVVGAVASTTVMGAGVADAGTMPKAAMQYQDTPKGKAQCSICAQFI----  64

Query  199   NLDHSGCKLYPGDDEICQNCYCNGFVQKA  285
                HS CK+  G+  I  N +C  F  K+
Sbjct  65    -APHS-CKVVAGN--ISPNGWCIAFAPKS  89
```

Figure S12 The BLAST alignment of predicted iron oxidation related genes in FKB7 against well-known associated genes in well-known iron oxidizers. The iron oxidation associated genes including those encoding cyc1/rus/cyc2 in *Acidithiobacillus ferrooxidans*, iro in *Acidithiobacillus ferrivorans*, cox operon in *Thiobacillus prosperus*, *fox* gene cluster in *Sulfolobus metallicus*, *foxEYZ* of *Rhodobacter capsulatus* SB1003, Cyt572/Cyt579 in *Leptospirillum* spp., *PioAB* in *Rhodopseudomonas palustris* TIE-1 and *MtrAB* in *Shewanellas* spp. and *Geobacter* spp..

Table S1 Information of pre-assembly treatment of metagenomic and metatranscriptomic data

Items	Metagenomic data				Metatranscriptomic data	
	500 bp		2000 bp		300 bp	
	Total bases (Gb)	Read pairs (M)	Total bases (Gb)	Read pairs (M)	Total bases (Gb)	Read pairs (M)
Raw reads	94.6	479.7	10.6	53.6	8.7	44.0
Quality reads	80.2	408.4	7.0	35.9	3.8	19.2
High-abundance sequences [a]	68.7	350.8	2.6	13.8	-	-
Low-abundance sequences	11.3	57.6	4.3	22.1	-	-

[a] Quality reads were split into two components by their K-mer (K = 31) depth (see Supplementary methods for details). Reads with K-mer depth > 15 were treated as high-abundance sequences, and those with K-mer depth ≤ 15 were treated as low-abundance sequences.

Table S2 Statistics for *de novo* assembly of metagenomic and metatranscriptomic data with different parameters

		Metagenomic data [a]					Metatrans data [b]	Combined data [c]
		K=47	K=51	K=55	K=59	K=63		
Total bases [Mb]	Contigs > 500 bp	61	67	70	66	60	30	92
	Contigs > 3000 bp	6.5	7.8	11	9	7.7	14	29
Number of Contigs [d]	Contigs > 500 bp	65213	68053	67514	63600	58648	16718	69035
	Contigs > 3000 bp	1209	1535	2025	1797	1536	1108	4906
N50 [bp]	Contigs > 500 bp	901	973	1059	1044	1029	2597	1667
	Contigs > 3000 bp	5441	5103	4984	4898	4929	37518	6448

[a] Velvet assemblies were conducted on the range of K-mers for the metagenomic data.

[b] Metatrans is short for metatranscriptomic. Trinity was used to assemble the metatranscriptomic data.

[c] Both metagenomic (including different K-mers results) and metatranscriptomic assembly results were merged and reassembled using both Newbler and Minimus2 assembler.

[d] Only contigs were taken into consideration. Scaffolds were broken into contigs by one or more “N” to avoid potential chimeric scaffolds.

Table S3 General information of the 11 draft genomes assembled from quality metagenomic and metatranscriptomic data

Items	FKB1	FKB2	FKB3	FKB4	FKB5	FKB6	FKB7	FKA8	FKA9	FKA10	FKA11
In metagenome											
Total reads	1430915	985349	396146	1007516	4434308	696299	704167694	186565	516031	272150	178856
Percentage (%) [a]	0.20	0.14	0.06	0.14	0.62	0.98	98.59	0.03	0.07	0.04	0.03
In metatranscriptome											
Total reads	244581	53870	177724	396140	255390	428683	17877053	367	237	2324	33883
Percentage (%)	1.26	0.28	0.91	2.03	1.31	2.20	91.82	0.002	0.001	0.01	0.17
Predicted genes [b]											
Total number	2785	3011	2188	2640	3547	2662	2285	1414	1441	1208	1388
Mean length	818	821	848	864	895	829	815	820	798	816	709
Mean GC (%)	58.5	54.0	58.3	59.6	67.4	45.8	40.2	44.2	36.5	40.4	41.6
Hits to protein databases [c]											
NCBI-nr	2602(93.4)	2807(93.2)	2035(93.0)	2491(94.4)	3360(94.7)	2226(83.6)	1686(73.8)	1173(83.0)	1345(93.3)	1085(90.0)	1014(73.1)
KEGG	2543(91.3)	2604(86.5)	1634(74.7)	2081(78.8)	3291(92.8)	2163(81.2)	1550(67.8)	985(70.0)	937(65.0)	824(68.2)	938(67.6)
eggNOG	2541(91.2)	2590(86.0)	1622(74.1)	2063(78.1)	3289(92.7)	2151(80.8)	1530(67.0)	981(69.4)	928(64.4)	814(67.4)	931(67.1)

Abbreviation: eggNOG, evolutionary genealogy of genes: Non-supervised Orthologous Groups; COG, Clusters of Orthologous Groups of proteins; KEGG, Kyoto Encyclopedia of Genes and Genomes; KO, KEGG orthology; NCBI-nr, National Center for Biotechnology Information non-redundant.

[a] The percentage of reads in each draft genome were estimated by the ratio of reads observed in that bin and total reads in all the 11 bins.

[b] Protein-encoding genes were predicted for each draft genome using Genemark (Zhu *et al.*, 2010).

[c] Genes matching hits in different databases via BLASTx (e-value ≤ 10^{-5}), the proportions (%) to the total predicted genes in each genomes are shown in bracket.

Table S4 Indicator genes in each of the eight active AMD taxa

	Indicator gene	KO number	FKB1	FKB2	FKB3	FKB4	FKB5	FKB6	FKB7	FKA11
FKB1	Nitrogen fixation	K02586	0.15	-	-	0.12	-	-	-	-
	DNA polymerase III	K02337	0.83	0.14	-	0.17	0.10	0.09	0.25	-
	Phenylalanyl-tRNA synthetase	K01889, K01890	0.66	0.16	0.06	0.05	0.03	0.02	0.16	-
	S-adenosylmethionine decarboxylase	K01611	2.88	-	-	0.19	0.68	1.26	-	-
	L-lactate dehydrogenase	K00016	0.07	-	-	-	-	0.05	-	-
	Preprotein translocase	K03071, K03210	0.40	0.06	-	-	0.05	0.02	0.06	-
	Host factor-I protein	K03666	0.39	0.06	-	-	-	0.00	0.07	-
	Ferredoxin--NAD+ reductase	K00529	0.30	-	-	-	-	-	-	-
	Pyruvate dehydrogenase E1 component	K00161	0.79	0.06	-	-	-	0.49	-	-
	Ribulose-bisphosphate carboxylase	K01601	1.26	0.26	0.16	0.08	0.01	-	0.54	-
FKB2	Flagellar assembly	K02397, K02406, K02422, K10941, K10942	0.06	0.88	0.14	0.07	-	-	-	-
	Cytochrome o ubiquinol oxidase	K02297, K02298	0.00	0.33	-	-	0.02	-	0.11	-
	Nitrate reductase	K00370, K00371	-	0.22	-	-	0.02	0.00	-	-
	Heterodisulfide reductase	K03388	-	0.16	-	-	-	-	-	-
	Capsular polysaccharide ABC transporter	K09688	0.02	0.11	-	-	-	-	-	-
	Cytochrome d ubiquinol oxidase	K00426	-	0.35	0.09	0.03	0.05	0.00	0.13	-
	Sulfur oxidation	K17227	-	0.08	-	-	-	-	-	-
	lysyl-tRNA synthetase	K04567	0.01	0.11	0.00	0.03	0.00	0.00	0.02	0.00
	Proline dehydrogenase/delta 1-pyrroline-5-carboxylate dehydrogenase	K13821	0.00	0.59	0.00	0.00	0.02	0.00	0.00	0.00
	Cytochrome c	K08738	0.25	1.85	0.00	0.00	0.24	0.00	0.20	0.00
FKB3	Pyruvate ferredoxin oxidoreductase	K00169, K00171	0.53	-	4.27	2.10	-	0.48	-	-
	Citryl-CoA synthetase	K15232, K15233	-	-	2.99	1.00	-	-	-	-
	Adenylylsulfate reductase	K00394	-	-	0.19	0.04	-	-	-	-
	Trehalose-6-phosphate synthase/phosphatase pathway	K00697, K01087, K16055	-	-	0.66	0.21	-	-	-	0.01
	Transaldolase	K00616	0.00	0.19	0.40	0.03	-	-	-	-
	Sirohydrochlorin ferrochelatase	K03794	-	-	0.22	0.08	-	0.02	0.01	-
	Cell cycle response regulator	K02488	0.01	-	0.55	0.07	0.02	0.17	0.28	-
	UDP-glucose 4-epimerase	K01784	0.02	0.06	0.94	0.16	0.06	0.08	0.06	0.05
	cheV; two-component system, chemotaxis family	K03415	-	0.07	0.19	0.05	-	-	-	-
	NADH-quinone oxidoreductase	K00332, K00333	-	-	0.77	0.11	0.17	0.02	0.16	-

Table S4 Indicator genes in each of the eight active AMD taxa (continued)

	Indicator gene	KO number	FKB1	FKB2	FKB3	FKB4	FKB5	FKB6	FKB7	FKA11
FKB4	Nitrite reductase (NADH)	K00363	-	-	0.03	0.09	-	-	0.00	0.00
	Pyruvate ferredoxin oxidoreductase	K00170	0.15	-	1.10	1.81	-	0.51	0.00	-
	Two-component system, NarL family	K07678	-	-	0.09	0.20	-	0.03	-	-
	Two-component system, NtrC family	K10125	-	-	-	0.12	0.00	-	-	-
	Formate--tetrahydrofolate ligase	K01938	-	-	0.05	0.45	0.02	0.04	0.12	-
	Acylphosphatase	K01512	-	-	-	0.16	-	-	-	0.07
	Pyruvate oxidase	K00158	-	-	-	0.09	-	-	-	-
	Proteasome-associated ATPase	K13527	-	-	0.31	0.46	-	-	-	-
	ATP diphosphatase	K04765	-	-	0.02	0.10	0.01	-	-	-
	Bidirectional [NiFe] hydrogenase diaphorase	K05587	-	-	0.28	0.36	-	0.00	-	-
FKB5	Spermidine/putrescine transport system	K11069, K11072	0.02	-	-	-	0.19	0.00	-	-
	Cytochrome c oxidase	K02259, K02261, K02274, K02276	-	-	-	-	0.79	-	-	0.23
	Carbon-monoxide dehydrogenase	K03518, K03519, K03520	-	-	-	-	1.43	-	-	0.15
	Acetyl-CoA acyltransferase	K00632	-	-	-	-	1.21	0.91	0.28	-
	Acetyl-CoA C-acetyltransferase	K00626	-	-	-	-	1.57	0.05	0.73	-
	NAD(P) transhydrogenase	K00325	-	-	-	-	0.12	-	0.05	-
	Ribose/Autoinducer 2/D-Xylose ABC transporter	K10439, K10440, K10441	-	0.07	-	-	0.43	-	-	-
	Sulfite oxidation	K00387	-	-	-	-	0.18	-	-	-
	Catalase-peroxidase	K03782	-	-	-	-	0.08	-	-	-
	Type VI secretion system	K11907	-	-	0.00	0.01	0.53	-	-	-
FKB6	Two-component system, chemotaxis family	K03406, K03407, K03412	0.04	0.02	0.72	0.36	0.07	2.23	-	-
	Phosphate ABC transporter	K02037, K02040	0.22	0.12	0.35	0.12	0.61	2.04	0.21	-
	Maltooligosaccharide/lactose/L-arabinose/maltose/ABC transporters	K10188, K15770	-	-	-	-	-	1.02	-	-
	Succinate dehydrogenase	K00240, K00241	-	-	-	-	0.29	0.82	0.46	-
	Phosphotransferase system (PTS), Fru family	K02770	-	-	-	-	0.00	0.51	-	-
	Glutathione peroxidase	K00432	-	0.02	-	-	-	0.61	0.14	-
	2-oxoglutarate ferredoxin oxidoreductase	K00174, K00175	-	-	-	-	-	0.48	-	-
	Superoxide dismutase, Fe-Mn family	K04564	-	0.10	-	-	0.12	0.69	0.03	-
	Glutamate dehydrogenase	K00260	-	-	-	-	-	1.51	-	-
	Galactokinase	K00849	-	-	-	-	-	0.96	-	-

Table S4 Indicator genes in each of the eight active AMD taxa (continued)

	Indicator gene	KO number	FKB1	FKB2	FKB3	FKB4	FKB5	FKB6	FKB7	FKA11
FKB7	Cytochrome c oxidase cbb3-type	K00404, K00405	-	-	0.90	0.29	-	-	1.74	-
	Sulfate reduction	K00390, K00955, K00957	0.02	-	-	-	0.07	0.11	1.24	-
	DNA polymerase III	K02338, K02341, K02342	0.06	0.04	0.13	0.18	0.41	0.14	1.37	-
	Formate dehydrogenase	K00123, K00124	-	-	-	-	0.74	0.19	1.18	-
	KdpD-KdpE (potassium transport) two-component regulatory system	K07646, K07667	0.04	0.00	0.00	0.02	0.05	0.01	0.75	-
	aceE; pyruvate dehydrogenase E1 component	K00163	-	-	-	-	0.18	-	0.54	-
	Pyrimidine deoxyribonuleotide biosynthesis	K00526, K00560	-	0.04	-	-	0.01	0.05	0.22	-
	Acetoacetyl-CoA reductase	K00023	-	-	-	-	0.04	0.05	0.24	-
	Phosphate acetyltransferase-acetate kinase pathway	K00625, K00925	0.20	0.07	-	0.13	0.14	-	0.69	-
	Oxygen-independent coproporphyrinogen III oxidase	K02495	0.01	0.09	0.10	0.05	0.02	0.04	0.70	-
FKA11	DNA-directed RNA polymerase	K03007, K03041, K03045, K03047, K13798	-	-	-	-	-	-	-	9.85
	Acetaldehyde dehydrogenase / alcohol dehydrogenase	K04072	-	-	-	-	-	0.02	-	0.62
	Elongation factor 1-alpha	K03231	-	-	-	-	-	-	-	0.95
	DNA polymerase	K02327	-	-	-	-	-	-	-	0.87
	Threonine synthase	K01733	-	0.15	0.36	0.22	0.04	0.06	0.09	3.48
	General transcription factors for RNA polymerase II	K03124	-	-	-	-	-	-	-	1.76
	S-adenosylmethionine synthetase	K00789	0.13	0.07	0.15	0.30	0.07	0.15	0.13	1.62
	Transcription initiation factor TFIIB	K03124	-	-	-	-	-	-	-	1.76
	Isocitrate dehydrogenase	K00031	0.34	0.16	-	0.40	0.88	1.69	0.18	3.70
	Replication factor A1	K07466	-	-	-	-	-	-	-	9.99

The shaded cells show the indicator genes identified for the taxon. Different KO numbers sharing the same function are combined and identified as one single indicator gene. Only the top ten indicator genes with the highest relative abundance of transcript pool in each taxon are shown. Non-integer numbers represent the relative abundance of genes in the corresponding taxon, "-" indicates the gene is not present or not expressed in the taxon.

Table S5 The relative transcriptional activity of genes involving in stress resistance in the active AMD taxa (corresponding to Figure 3b in the main text)

Stress	COG	Category	Functional description	FKB1	FKB2	FKB3	FKB4	FKB5	FKB6	FKB7	FKA11
	COG1657	I	Squalene cyclase (hopanoid biosynthesis)	1.45	0.45	0.86	0.75	1.12			
	COG2060	P	K+-transporting ATPase, A chain	0.18			0.42		0.82		2.64
	COG2216	P	K+-transporting ATPase, B chain	0.28			0.13	0.12	0.64		1.94
	COG2156	P	K+-transporting ATPase, C chain	0.15				0.15	0.25		
	COG1009	CP	Multisubunit Na+/H+ antiporter, MnhA subunit	0.52	0.19	0.22	0.21	0.60	0.36	0.34	0.82
Acid stress	COG0651	CP	Multisubunit Na+/H+ antiporter, MnhD subunit	0.24	0.28	0.12	0.27	1.75	0.14		0.20
	COG1055	P	Na+/H+ antiporter NhaD	0.17	0.64	0.22	0.23	0.66	0.22		
	COG0025	P	NhaP-type Na+/H+ and K+/H+ antiporters	0.13			0.11				
	COG3263	P	NhaP-type Na+/H+ and K+/H+ antiporters			0.27			0.26		
	COG1982	E	arginine/lysine/ornithine decarboxylase				0.30		0.40	0.55	
	COG1166	E	arginine decarboxylase		0.27						
	COG0845	M	Membrane-fusion protein	0.17	0.26	0.84	0.57	0.23	0.07	0.75	
	COG1538	MU	Outer membrane protein	0.43	0.79	0.83	1.22	0.82		0.73	
	COG0798	P	Arsenite efflux pump ACR3 and related permeases						0.05	0.71	
	COG0861	P	Membrane protein TerC, possibly involved in tellurium resistance						0.74	0.33	
	COG1275	P	Tellurite resistance protein and related permeases	1.87	0.90						
	COG1108	P	ABC-type Mn2+/Zn2+ transport systems		0.22			0.78			
	COG0474	P	Cation transport ATPase	0.19	0.42	0.24	0.06	0.08	0.01	0.31	0.70
Heavy metal stress	COG2217	P	Cation transport ATPase	0.33	0.56	1.05	1.12	0.25	0.40	1.86	
	COG2059	P	Chromate transport protein ChrA					0.78		1.09	
	COG1230	P	Co/Zn/Cd efflux system component	0.23	0.35			0.37	0.84	0.68	
	COG0735	P	Fe2+/Zn2+ uptake regulation proteins	4.68	2.39	2.38	0.58	1.66	1.97	2.77	
	COG0672	P	High-affinity Fe2+/Pb2+ permease				0.73				
	COG0598	P	Mg2+ and Co2+ transporters	0.31	0.25						
	COG0053	P	Predicted Co/Zn/Cd cation transporters	0.81	0.44	0.07	0.32	0.10	0.08		
	COG3696	P	Putative silver efflux pump	0.17	0.09	0.33	0.15	0.12		0.24	
	COG0841	V	Cation/multidrug efflux pump	0.32	0.22	0.67	0.32	0.36	0.07	0.69	
	COG0376	P	Catalase (peroxidase I)					0.31			
	COG0386	O	Glutathione peroxidase		0.33				9.37	1.80	
	COG0450	O	Peroxiredoxin	0.83	9.23	1.77	0.89	0.43	0.17	0.97	3.12
Oxidative stress	COG0605	P	Superoxide dismutase		4.66			2.28	13.92	0.57	
	COG0695	O	putative glutaredoxin	0.30	0.84			2.00		0.29	
	COG1858	P	cytochrome-c peroxidase	0.74			0.38	0.64			
	COG2077	O	Redoxin domain protein						7.79		
	COG3253	S	Chlorite dismutase				1.38		0.59		

Table S6 The relative transcriptional activity of physiologically relevant genes in the active AMD taxa

	KO	Gene	Functional description	Relative expression activity							
				FKB1	FKB2	FKB3	FKB4	FKB5	FKB6	FKB7	FKA11
**Carbon fixation**											
CBB cycle	K01601	*rbcL*	Ribulose 1,5-bisphosphate carboxylase, large subunit	1.42	1.53	0.45	0.19			3.05	
	K01602	*rbcS*	Ribulose 1,5-bisphosphate carboxylase, small subunit	1.54	6.82					3.70	
	K00855	*prkB*	Phosphoribulokinase	0.74	1.80					0.60	
novel rTCA cycle (*Leptospirillum* spp.)	K00244	*frdA*	Fumarate reductase, flavoprotein subunit			1.53	0.45				
	K15232	*ccsA*	citryl-CoA synthetase large subunit			4.22	1.66				
	K15233	*ccsB*	citryl-CoA synthetase small subunit			15.47	7.68				
	K15234	*ccl*	citryl-CoA lyase			0.83	0.65				
	K00169	*porA*	pyruvate ferredoxin oxidoreductase, alpha subunit			1.73	2.76				
	K00170	*porB*	pyruvate ferredoxin oxidoreductase, beta subunit			1.52	1.23				
	K00171	*porD*	pyruvate ferredoxin oxidoreductase, delta subunit			2.42					
	K00172	*porG*	pyruvate ferredoxin oxidoreductase, gamma subunit			2.49	1.43				
**Nitrogen metabolism**											
Nitrogen fixation	K02586	*nifD*	nitrogenase molybdenum-iron protein alpha chain	2.08			0.58				
	K02591	*nifK*	nitrogenase molybdenum-iron protein beta chain				0.20				
	K02588	*nifH*	nitrogenase iron protein	0.42			0.61				
Nitrogen resources uptake	K03320	*nrgA*	ammonium transporter	0.19	0.60		0.23	1.80	0.20	0.19	
	K08717	*utp*	urea transporter								
	K02575	*narK*	nitrate/nitrite transporter		0.14					0.37	
Assimilatory nitrate/nitrite reduction	K00367	*narB*	ferredoxin-nitrate reductase								
	K10534	NR	nitrate reductase (NAD(P)H)								
	K00372	*nasA*	assimilatory nitrate reductase catalytic subunit		0.07			0.04		0.17	
	K00360	*nasB*	assimilatory nitrate reductase electron transfer subunit								
	K00366	*nirA*	ferredoxin-nitrite reductase			0.36					
	K17877	NIT-6	nitrite reductase (NAD(P)H)								

Table S6 The relative transcriptional activity of physiologically relevant genes in the active AMD taxa (continued)

	KO	Gene	Functional description	Relative expression activity							
				**FKB1**	**FKB2**	**FKB3**	**FKB4**	**FKB5**	**FKB6**	**FKB7**	**FKA11**
Dissimilatory nitrate/nitrite reduction	K00370	*narG*	nitrate reductase 1, alpha subunit		0.30			0.09	0.01		
	K00371	*narH*	nitrate reductase 1, beta subunit		0.22			0.06			
	K00374	*narI*	nitrate reductase 1, gamma subunit		0.22			0.00	0.70		
	K00373	*narJ*	nitrate reductase 1, delta subunit		0.33						
	K02567	*napA*	periplasmic nitrate reductase NapA								
	K02568	*napB*	cytochrome c-type protein NapB								
	K00362	*nirB*	nitrite reductase (NAD(P)H) large subunit					0.04		0.11	
	K00363	*nirD*	nitrite reductase (NAD(P)H) small subunit			2.82	3.52			0.12	
	K03385	*nrfA*	nitrite reductase (cytochrome c-552)								
	K15876	*nrfH*	cytochrome c nitrite reductase small subunit								
Glutamine synthetase/glutamate synthase	K01915	*glnA*	glutamine synthetase	2.45	1.43		0.61	1.05	1.24	0.53	0.47
	K00265	*gltB*	glutamate synthase (NADPH/NADH) large chain	1.74	0.45	0.84	0.81	2.71	0.13	1.29	
	K00266	*gltD*	glutamate synthase (NADPH/NADH) small chain	1.16	0.23	4.24	0.87	3.54	0.83	1.09	1.80
Urea metabolism	K03190	*ureD*	urease accessory protein							0.37	
	K01430	*ureA*	urease gamma subunit							0.35	
	K01429	*ureB*	urease beta subunit							0.17	
	K03192	*ureJ*	urease accessory protein							0.14	
	K01428	*ureC*	urease alpha subunit							0.28	
	K03187	*ureE*	urease accessory protein							0.27	
	K03188	*ureF*	urease accessory protein							0.33	
	K03189	*ureG*	urease accessory protein							0.46	
**Iron oxidation**		*rus*	rusticyanin	28.49							
		c572	cytochrome 572			1.04	1.99				
		c579	cytochrome 579			7.59					

Table S6 The relative transcriptional activity of physiologically relevant genes in the active AMD taxa (continued)

	KO	Gene	Functional description	Relative expression activity							
				FKB1	FKB2	FKB3	FKB4	FKB5	FKB6	FKB7	FKA11
**Sulfur metabolism**											
Sulfur oxidation		SQR	sulfide quinone reductase	2.01	0.14			2.01			
	K17222	*soxA*	sulfur oxidation protein SoxA		0.46						
	K17223	*soxX*	sulfur-oxidizing protein SoxX		1.91						
	K17224	*soxB*	sulfur oxidation protein SoxB		0.22						
	K17226	*soxY*	sulfur oxidation protein SoxY		0.67						
	K17227	*soxZ*	sulfur oxidation protein SoxZ		2.15						
		TQO	thiosulfate:quinone oxidoreductase small subunit	0.79	1.36						
		TTH	tetrathionate hydrolase	10.06	0.48						
	K16952	SOR	sulfur oxygenase reductase								
	K17725	ETHE1	sulfur dioxygenase		1.38			3.42	1.59		
Assimilatory sulfur reduction	K13811	PAPSS	3'-phosphoadenosine 5'-phosphosulfate synthase								
	K00958	*sat*	sulfate adenylyltransferase			1.08	0.82		0.83		
	K00955	*cysNC*	bifunctional enzyme CysN/CysC					0.22		2.28	
	K00957	*cysD*	sulfate adenylyltransferase subunit 2	0.15				0.28		3.01	
	K00956	*cysN*	sulfate adenylyltransferase subunit 1	0.23							
	K00860	*cysC*	adenylylsulfate kinase							1.74	
	K00390	*cysH*	phosphoadenosine phosphosulfate reductase						2.52	3.21	
	K00380	*cysJ*	sulfite reductase (NADPH) flavoprotein alpha-component					0.14			
	K00381	*cysI*	sulfite reductase (NADPH) hemoprotein beta-component					0.09	0.82	2.67	
	K00392	*sir*	sulfite reductase (ferredoxin)								
Dissimilatory sulfate reduction	K00956	*cysN*	sulfate adenylyltransferase subunit 1	0.23							
	K00957	*cysD*	sulfate adenylyltransferase subunit 2	0.15				0.28		3.01	
	K00958	*sat*	sulfate adenylyltransferase			1.08	0.82		0.83		
	K00394	*aprA*	adenylylsulfate reductase, subunit A			2.30	0.61				
	K00395	*aprB*	adenylylsulfate reductase, subunit B			0.63	0.31				
	K11180	*dsrA*	sulfite reductase alpha subunit								
	K11181	*dsrB*	sulfite reductase beta subunit								

Table S7 The relative transcriptional activity of transporting protein coding genes for dissolved organic carbon (DOC) resources in the active AMD taxa

DOC	COG	FKB1	FKB2	FKB3	FKB4	FKB5	FKB6	FKB7	FKA11	Functional description
Amino Acids	COG0834					0.31				ABC-type amino acid transport/signal transduction systems, periplasmic component
	COG1126					0.33				ABC-type polar amino acid transport system, ATPase component
	COG0765					0.10				ABC-type amino acid transport system, permease component
	COG0531	0.28	0.40	0.25	0.21	0.70	0.32	3.38	1.67	Amino acid transporters
	COG0683				0.38	1.40		1.36		ABC-type branched-chain amino acid transport systems, periplasmic component
	COG0559					0.38		0.35		Branched-chain amino acid ABC-type transport system, permease components
	COG0410					0.69	0.46	0.84		ABC-type branched-chain amino acid transport systems, ATPase component
	COG4177					0.52		0.66		ABC-type branched-chain amino acid transport system, permease component
	COG0411					0.70		0.60		ABC-type branched-chain amino acid transport systems, ATPase component
	COG0747	0.22	0.82	0.23	0.50	0.24	6.21	0.37		ABC-type dipeptide transport system, periplasmic component
	COG0601	0.27	0.64	0.44	0.14	0.10	1.70	0.29	2.30	ABC-type dipeptide/oligopeptide/nickel transport systems, permease components
	COG1173	0.19	0.27	0.22	0.14	0.12	1.12	0.36	2.24	ABC-type dipeptide/oligopeptide/nickel transport systems, permease components
	COG4608	0.36	0.80	0.54	0.43		2.56		2.90	ABC-type oligopeptide transport system, ATPase component
	COG0444	0.22	0.83	0.74	0.40		1.64		2.10	ABC-type dipeptide/oligopeptide/nickel transport system, ATPase component
	COG4166			0.27			0.15			ABC-type oligopeptide transport system, periplasmic component
Polyamines	COG0687					1.57				Spermidine/putrescine-binding periplasmic protein
	COG3842	0.59				1.55	0.70	0.53		ABC-type spermidine/putrescine transport systems, ATPase components
	COG1176					0.82				ABC-type spermidine/putrescine transport system, permease component I
	COG1177					1.55	0.19			ABC-type spermidine/putrescine transport system, permease component II
Compatible Solutes	COG2113					1.12				ABC-type proline/glycine betaine transport systems, periplasmic components
	COG4176					0.39				ABC-type proline/glycine betaine transport system, permease component
	COG0591					0.60	4.72	0.64		Na+/proline symporter
	COG4175					0.45				ABC-type proline/glycine betaine transport system, ATPase component
	COG1125						0.30			ABC-type proline/glycine betaine transport systems, ATPase components

Table S7 The relative transcriptional activity of transporting protein coding genes for dissolved organic carbon (DOC) resources in the active AMD taxa (continued)

DOC	COG	FKB1	FKB2	FKB3	FKB4	FKB5	FKB6	FKB7	FKA11	Functional description
Lipids	COG0580	1.40	0.29			1.49				Glycerol uptake facilitator and related permeases (Major Intrinsic Protein Family)
	COG2067	1.88	8.12			2.47		3.30		Long-chain fatty acid transport protein
	COG2867	0.86	2.17			0.88		0.55		Oligoketide cyclase/lipid transport protein
Carbohydrates	COG1653		0.13			0.41	2.22			ABC-type sugar transport system, periplasmic component
	COG1879		0.98			0.77				ABC-type sugar transport system, periplasmic component
	COG3839		0.28			0.25				ABC-type sugar transport systems, ATPase components
	COG1175		0.60			0.22	0.26		0.88	ABC-type sugar transport systems, permease components
	COG0395		0.50			0.15	0.23		0.54	ABC-type sugar transport system, permease component
	COG1129		0.28			0.73				ABC-type sugar transport system, ATPase component
	COG2211					0.14				Na+/melibiose symporter and related transporters
	COG0738						0.27			Fucose permease
	COG1134	2.53				0.76	0.25	1.91		ABC-type polysaccharide/polyol phosphate transport system, ATPase component
	COG1682	2.55	7.62			1.30	0.10	1.51		ABC-type polysaccharide/polyol phosphate export systems, permease component
	COG1762	2.62					3.29	1.31		Phosphotransferase system mannitol/fructose-specific IIA domain (Ntr-type)
	COG1925	0.76	1.42			2.25	8.59	1.52		Phosphotransferase system, HPr-related proteins
	COG2893	0.30				0.97				Phosphotransferase system, mannose/fructose-specific component IIA
	COG1172		0.80			0.59				Ribose/xylose/arabinose/galactoside ABC-type transport systems, permease components
	COG4214					0.90				ABC-type xylose transport system, permease component
	COG2182						4.60		1.45	Maltose-binding periplasmic proteins/domains
	COG3833						0.95			ABC-type maltose transport systems, permease component
Carboxylic Acids	COG1593					0.23		0.58		TRAP-type C4-dicarboxylate transport system, large permease component
	COG1638					0.13		0.61		TRAP-type C4-dicarboxylate transport system, periplasmic component
	COG2358					1.22				TRAP-type uncharacterized transport system, periplasmic component
	COG0471				0.10					Di- and tricarboxylate transporters
	COG0651	0.23	0.21	0.12	0.27	1.80	0.14		0.20	Formate hydrogenlyase subunit 3/Multisubunit Na+/H+ antiporter, MnhD subunit
	COG1620						0.30	0.33		L-lactate permease
	COG3090							0.70		TRAP-type C4-dicarboxylate transport system, small permease component

The relative transcriptional activity of DOC transporting protein coding genes detected in each taxon is shown. The total numbers of associated genes detected in the taxa are 16, 19, 8, 9, 40, 24, 21 and 9, respectively.

Table S8 Finished complete or near-complete genomes for each phylogenetic order used as reference for core COGs analysis

Order	Family	Genus	Strain	COG clusters [a]	No. of core COGs [b]
Acidithiobacillales	*Acidithiobacillaceae*	*Acidithiobacillus*	*Acidithiobacillus caldus* ATCC 51756	1289	995
	Acidithiobacillaceae	*Acidithiobacillus*	*Acidithiobacillus caldus* SM-1	1374	
	Acidithiobacillaceae	*Acidithiobacillus*	*Acidithiobacillus ferrivorans* SS3	1430	
	Acidithiobacillaceae	*Acidithiobacillus*	*Acidithiobacillus ferrooxidans* ATCC 23270	1366	
	Acidithiobacillaceae	*Acidithiobacillus*	*Acidithiobacillus ferrooxidans* ATCC 53993	1362	
	Acidithiobacillaceae	*Acidithiobacillus*	*Acidithiobacillus* sp. GGI-221	1320	
Nitrospirales	*Nitrospiraceae*	*Leptospirillum*	Candidatus *Nitrospira defluvii*	1587	661
	Nitrospiraceae	*Leptospirillum*	*Leptospirillum ferriphilum* ML-04	1051	
	Nitrospiraceae	*Leptospirillum*	*Leptospirillum rubarum*	1077	
	Nitrospiraceae	*Leptospirillum*	*Leptospirillum ferrodiazotrophum*	1094	
	Nitrospiraceae	*Leptospirillum*	*Thermodesulfovibrio yellowstonii* DSM 11347	1168	
Rhodospirillales	*Acetobacteraceae*	*Acetobacter*	*Acetobacter pasteurianus* IFO 3283-01	1370	762
	Acetobacteraceae	*Acidiphilium*	*Acidiphilium cryptum* JF-5	1635	
	Acetobacteraceae	*Gluconacetobacter*	*Gluconacetobacter diazotrophicus* Pal 5,DSM_5601	1606	
	Acetobacteraceae	*Gluconobacter*	*Gluconobacter oxydans* 621H	1339	
	Acetobacteraceae	*Granulibacter*	*Granulibacter bethesdensis* CGDNIH1	1414	
	Rhodospirillaceae	*Azospirillum*	*Azospirillum* sp. B510	2025	
	Rhodospirillaceae	*Magnetospirillum*	*Magnetospirillum magneticum* AMB-1	1649	
	Rhodospirillaceae	*Rhodocista*	*Rhodocista centenaria* SW	1771	
	Rhodospirillaceae	*Rhodospirillum*	*Rhodospirillum rubrum* S1, ATCC 11170	1782	
unknown	unknown	unknown	Candidatus *Micrarchaeum acidiphilum*	520	278
	unknown	unknown	Candidatus *Parvarchaeum acidiphilum*	404	
	unknown	unknown	Candidatus *Parvarchaeum acidophilus*	422	
Nitrosomonadales	*Nitrosomonadaceae*	*Nitrosospira*	*Nitrosospira multiformis* ATCC 25196	1447	1044
	Nitrosomonadaceae	*Nitrosomonas*	*Nitrosomonas europaea* ATCC 19718	1377	
	Nitrosomonadaceae	*Nitrosomonas*	*Nitrosomonas eutropha* C91	1377	
	Nitrosomonadaceae	*Nitrosomonas*	*Nitrosomonas* sp. AL212	1448	
	Nitrosomonadaceae	*Nitrosomonas*	*Nitrosomonas* sp. Is79A3	1523	
Bacillales	*Alicyclobacillaceae*	*Alicyclobacillus*	*Alicyclobacillus acidocaldarius* DSM 446	1381	981
	Paenibacillaceae	*Brevibacillus*	*Brevibacillus brevis* NBRC 100599	1836	
	Paenibacillaceae	*Paenibacillus*	*Paenibacillus* sp. JDR-2	1790	
	Bacillaceae	*Bacillus*	*Bacillus halodurans* C-125	1742	
	Bacillaceae	*Bacillus*	*Bacillus clausii* KSM-K16	1704	
Thermoplasmatales	*Picrophilaceae*	*Picrophilus*	*Picrophilus torridus* DSM 9790	910	728
	Thermoplasmataceae	*Thermoplasma*	*Thermoplasma acidophilum* DSM 1728	887	
	Thermoplasmataceae	*Thermoplasma*	*Thermoplasma volcanium* GSS1	896	
	Ferroplasmaceae	*Ferroplasma*	*Ferroplasma acidarmanus* fer1	913	

a. COG clusters stands for number of genes matching hits via BLASTx to COG database.

b. Core COGs were defined as number of the COGs shared by the corresponding pan-genome (as listed genomes in the table).
