# Supplementary figures and images for: Ecological roles of dominant and rare prokaryotes in acid mine drainage revealed by metagenomics and metatranscriptomics
Source: ISME J. 2014 Nov 7;9(6):1280–94. doi: 10.1038/ismej.2014.212 (PMC4438317; doi:10.1038/ismej.2014.212)

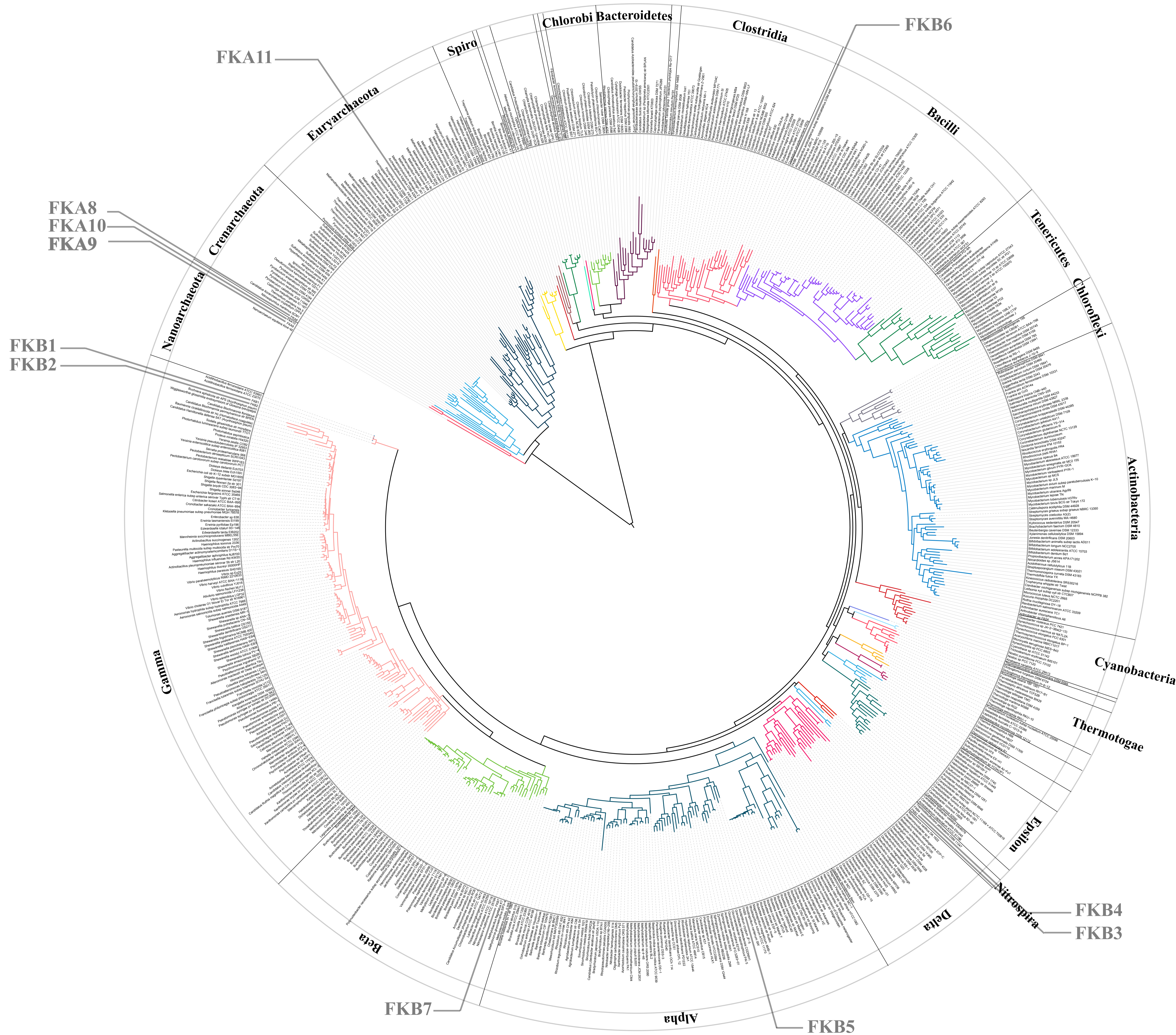

Supplement: Supplementary Figure 5 [file ismej2014212x2.pdf]
